# Effects of Near Wall Modeling in the Improved-Delayed-Detached-Eddy-Simulation (IDDES) Methodology

**DOI:** 10.3390/e20100771

**Published:** 2018-10-08

**Authors:** Rohit Saini, Nader Karimi, Lian Duan, Amsini Sadiki, Amirfarhang Mehdizadeh

**Affiliations:** 1 Civil and Mechanical Engineering Department, School of Computing and Engineering, University of Missouri-Kansas City, Kansas City, MO 64110, USA; 2 School of Engineering, University of Glasgow, Glasgow G12 8QQ, UK; 3 Department of Mechanical and Aerospace Engineering, Missouri University of Science and Technology, Rolla, MO 65409, USA; 4 Department of Mechanical Engineering, Institute of Energy and Power Plant Technology, Technische Universität Darmstadt, 64289 Darmstadt, Germany

**Keywords:** hybrid (U)RANS-LES, IDDES methodology, attached and separated flows

## Abstract

The present study aims to assess the effects of two different underlying RANS models on overall behavior of the IDDES methodology when applied to different flow configurations ranging from fully attached (plane channel flow) to separated flows (periodic hill flow). This includes investigating prediction accuracy of first and second order statistics, response to grid refinement, grey area dynamics and triggering mechanism. Further, several criteria have been investigated to assess reliability and quality of the methodology when operating in scale resolving mode. It turns out that irrespective of the near wall modeling strategy, the IDDES methodology does not satisfy all criteria required to make this methodology reliable when applied to various flow configurations at different Reynolds numbers with different grid resolutions. Further, it is found that using more advanced underlying RANS model to improve prediction accuracy of the near wall dynamics results in extension of the grey area, which may delay the transition to scale resolving mode. This systematic study for attached and separated flows suggests that the shortcomings of IDDES methodology mostly lie in inaccurate prediction of the dynamics inside the grey area and demands further investigation in this direction to make this methodology capable of dealing with different flow situations reliably.

## 1. Introduction

High Reynolds number flows are a classical research theme that retains its vitality at several levels from real-world applications, through physical and computational modeling, up to rigorous mathematical analysis. The main reason for the sustained relevance of this topic is in the ubiquity of such flows in practical situations, such as blood flow in large caliber vessels, various energy systems, aerodynamics, combustion systems, to name only a few. Numerical simulation of high Reynolds number flows is supposed to serve the purpose of providing necessary data for design and optimization. However, modeling high Reynolds number flows is immensely challenging due to the complex interaction among disparate turbulent length scales associated with different regimes in these flows. Advanced modeling strategies are needed to describe the interaction between different flow regions, e.g., surface viscous layers and outer turbulent flow regions in wall-bounded turbulent flows.

Reynolds-Averaged Navier–Stokes (RANS) models are generally used to simulate stationary high Reynolds number turbulent flows with industrial applications. However, it is becoming increasingly clear that there is a need to capture unsteady dynamics of the complex turbulent flows where classical RANS models either cannot provide necessary data (e.g., acoustics simulations where the turbulence generates noise sources, which cannot be extracted accurately from RANS simulations) or they are not accurate enough even in the first order statistics (e.g., strongly separated flows such as flow past a building and a re-entry vehicle). Unsteady extensions of RANS models (denoted as (U)RANS) attempt to capture some level of unsteady dynamics. However, because (U)RANS methods are not designed to capture integral-scale dynamics, Large Eddy Simulation (LES) is sometimes needed to capture essential energetic unsteady dynamics in complex flows. Unfortunately, LES is not feasible for many engineering applications due to the high computational cost associated with grid refinement to resolve the energy-containing eddies appropriately. This limitation becomes immense to capture near-wall dynamics. In the vicinity of the walls, the LES philosophy of resolving energy-containing vortical structures requires grid refinement probably close to the Direct Numerical Simulation (DNS) level, which makes LES prohibitively expensive to apply to wall-bounded flows at high Reynolds number.

Recognizing the limitations of the classical RANS/(U)RANS and LES and in search for more efficient solution methods for practical applications, the CFD community has turned its attention to hybrid (U)RANS-LES and wall-modeled LES approaches as alternative strategies for complex turbulent flow with high Reynolds numbers. The primary goal of a hybrid (U)RANS-LES/wall-modeled LES approach is to achieve time-dependent and three-dimensional space-resolved simulation of large-scale structures, which describe the turbulence dynamics at an affordable cost, while near-wall dynamics are accurately modeled. Several hybrid (U)RANS-LES approaches have been proposed within the last two decades. These included detached eddy simulation [1], scale-adaptive simulation [2], partially-averaged Navier–Stokes [3], etc. Among them, the Detached Eddy Simulation (DES) developed originally by Spalart [1], including its variants Delayed Detached Eddy Simulation (DDES) [4] and Improved Delayed Detached Eddy Simulation (IDDES) [5], has attracted the most attention due to its simplicity in implementation, and it is widely used to simulate high Reynolds number flows relevant for industrial applications [6].

The first version of DES was based on a modified transport equation for turbulent eddy viscosity ($\nu_{t}$) that uses distance from the wall as the RANS length scale. Local grid refinement is used to alter the length scale away from the wall to drive the model into a scale-resolving mode. However, this approach faced several practical issues, in particular, Grid-Induced Separation (GIS), Model Stress Depletion (MSD) and Log-layer Mismatch (LMM), which were discussed in [7]. DDES [4] and IDDES [5] have consequently been proposed to mitigate these issues. IDDES features Wall-Modeled-LES (WMLES) capabilities, depending on inflow condition and, therefore, includes more empiricism. The IDDES methodology can basically be combined with various RANS models to form a hybrid approach [6]. In the present work, focus will be on Spalart–Allmaras IDDES (uses distance from the wall to provide RANS length scale [5]) and *k*-ω-SSTIDDES (uses the two-equation model to provide the RANS length scale [8]). In particular, the effect of the underlying RANS model in overall model behavior will be investigated. This includes response to grid refinement, prediction accuracy, grey-area dynamics and the triggering mechanism. Toward this end, Spalart–Allmaras (S-A) IDDES and *k*-ω-SST IDDES will be applied to different configurations ranging from fully-attached to complex separated flows.

The paper is organized as follows: In the next section, the IDDES formulations will be briefly presented and discussed. In Section 3, an overview of the test cases is provided. Section 4 and Section 5 are dedicated to present quality assessment criteria and the numerical approach. Section 6 will present and discuss the results obtained from the IDDES methodology using different near-wall modeling strategies. Section 7 concludes the paper with a summary, conclusion and outlook.

## 2. Improved Delayed Detached Eddy Simulation Methodology

In this section, a brief description of the governing transport equations of S-A IDDES and *k*-ω-SST IDDES models along with the triggering mechanism involved in the IDDES methodology will be presented.

### 2.1. Spalart–Allmaras IDDES

S-A IDDES is defined based on the transport equation for modified eddy viscosity ($\tilde{\nu }$) and is given as follows:

(1) $$\frac{\partial \tilde{\nu }}{\partial t}+U_{i}\frac{\partial \tilde{\nu }}{\partial x_{j}}=c_{b1}\tilde{S}\tilde{\nu }+\frac{1}{\sigma }\left[▽.\left(\tilde{\nu }▽\tilde{\nu }\right)+c_{b2}{\left(▽\tilde{\nu }\right)}^{2}\right]-c_{w1}f_{w}\left(\tilde{r}{\left(\frac{\tilde{\nu }}{l_{IDDES}}\right)}^{2}\right)$$

where the turbulent eddy viscosity is defined as $\nu_{t}=f_{v1}\tilde{\nu }$. Functions $f_{v1}$ and $f_{w}$ are introduced for near-wall corrections in the case of finite and high Reynolds number flows, respectively. $\tilde{S}$ is the strain rate tensor, and $\tilde{r}$ is the non-dimensional term defined as $\nu_{t}/\left(\tilde{S}\kappa^{2}{d_{w}}^{2}\right)$, where κ and $d_{w}$ are the von-Karman constant and distance from the wall. σ, $c_{b1}$, $c_{b2}$ and $c_{w}$ are the model constants imported from the original Spalart–Allmaras (S-A) model [9]. A complete description of the model is provided in Shur et al. [5]. The $l_{IDDES}$ term is a modified length scale responsible for triggering to a scale-resolving mode and will be discussed in Section 2.3.

### 2.2. k-ω-SST IDDES

*k*-ω-SST IDDES employs a modified version of *k*-ω-SST model to improve near-wall prediction and is defined as below:

(2) $$\frac{\partial k}{\partial t}+▽.\left(\tilde{U}k\right)=▽.\left[\left(\nu +\sigma_{k}\nu_{t}\right)▽k\right]+P_{k}-\sqrt{k^{3}}/l_{IDDES},$$

(3) $$\frac{\partial \omega }{\partial t}+▽.\left(\tilde{U}\omega \right)=▽.\left[\left(\nu +\sigma_{\omega }\nu_{t}\right)▽\omega \right]+2\left(1-F_{1}\right)\sigma_{\omega 2}\frac{▽k.▽\omega }{\omega }+\alpha \frac{1}{\nu_{t}}P_{k}-\beta \omega^{2},$$

where blending function $F_{1}$ and model constants (α, $\sigma_{k}$, $\sigma_{\omega }$, $\sigma_{\omega 2}$ and β) are imported from the original *k*-ω-SST model [10]. It should be noted that within *k*-ω-SST IDDES, only the destruction term in the *k*-equation is modified by introducing the $l_{IDDES}$ term, whereas the ω equation remains unchanged. Similar to S-A IDDES, $l_{IDDES}$ is responsible for triggering a transition from (U)RANS mode into a scale-resolving mode.

### 2.3. Triggering Mechanism

The goal in the IDDES methodology is to trigger a transition from (U)RANS to a scale-resolving mode, depending on a criterion based on the turbulent length scale. In this context, the $l_{IDDES}$ term is applied to the destruction term in the modified eddy viscosity, $\tilde{\nu }$ (Equation (1)) and turbulent kinetic energy, k (Equation (2)), transport equations. The intention is to increase dissipation (reduce the level of turbulent eddy viscosity) as we transverse away from the wall to trigger a transition to scale-resolving mode. The $l_{IDDES}$ term is defined as follows:

(4) $$l_{IDDES}=\tilde{f_{d}}\left(1+f_{e}\right)l_{RANS}+\left(1-\tilde{f_{d}}\right)l_{LES},$$

where $l_{RANS}$ for S-A IDDES is simply distance from the wall ($d_{w}$) and for *k*-ω-SST IDDES corresponds to $k^{2}/\left(C_{\mu }\omega \right)$, with $C_{\mu }=0.09$. In scale-resolving mode, $l_{LES}$ for S-A IDDES is defined as $C_{DES}\psi ▵$, where $C_{DES}=0.65$ and ψ is the low Reynolds number correction, which accommodates near-wall corrections, as discussed in [4]. In case of *k*-ω-SST IDDES, ψ equals one, and $C_{DES}$ is calculated algebraically as below:

(5) $$C_{DES}=C_{DES1}\cdot F_{1}+C_{DES2}\cdot \left(1-F_{1}\right),$$

with $C_{DES1}$ = 0.78, $C_{DES2}$ = 0.61 and $F_{1}$ is calculated as per the original *k*-ω-SST turbulence model [10]. Filter width or the characteristic cut-off length scale (▵), used in calculating LES length scale, is a piece-wise function containing wall-distance dependency and local cell dimension information:

(6) $$▵=min(max\left[C_{w}d_{w},C_{w}h_{max},h_{wn}\right],h_{max}),$$

where $C_{w}$ is an empirical constant and is 0.15, $h_{max}$ is the maximum of the local cell size in streamwise, wall-normal and lateral directions and $h_{wn}$ is the wall-normal grid spacing. The $\tilde{f_{d}}$ function in Equation (4) includes a set of blending functions responsible for switching from (U)RANS mode (defined by $\tilde{f_{d}}$ = 1) to scale-resolving mode (defined by $\tilde{f_{d}}$ = 0). However, transition from (U)RANS to scale-resolving mode does occur through an intermediate area called the grey area, where $0<\tilde{f_{d}}<1$. The function $\tilde{f_{d}}$ is defined as follows:

(7) $$\tilde{f_{d}}=max\left(1-f_{d},f_{B}\right).$$

$f_{d}$ is called the delaying function and is defined as below:

(8) $$f_{d}=1-\tanh\left[8\left({r_{d}}^{3}\right)\right],$$

where the $r_{d}$ term is borrowed from the original S-A model [9].

To provide a remedy to the log layer mismatch at the interface of (U)RANS and scale-resolving region, the IDDES methodology includes wall modeling capability called Wall-Modeled LES (WMLES). The WMLES branch of IDDES is intended to be active if turbulent inflow content is provided and the grid is fine enough to resolve the dominant vortical structures in the boundary layer. Under appropriate conditions for WMLES operation, the $l_{IDDES}$ defined in Equation (4) is modified to $l_{WMLES}$ as follows:

(9) $$l_{WMLES}=f_{B}\left(1+f_{e}\right)l_{RANS}+\left(1-f_{B}\right)l_{LES},$$

where the blending function $f_{B}$ is purely grid dependent and is based on the distance from the wall and the local maximum cell edge length. $f_{B}$ is described as follows:

(10) $$f_{B}=min\left[2exp\left(-9\alpha^{2}\right),1.0\right],$$

where the grid-dependent parameter α is calculated as α = $0.25-(d_{w}/h_{max}$). $f_{B}$ varies from zero to one and should provide a rapid transition from (U)RANS mode to scale-resolving mode within the range of wall distance 0.5$h_{max}$ < $d_{w}$ < $h_{max}$. Another empirical function called elevating function $f_{e}$, included in Equation (9), helps in preventing the excessive reduction of the Reynolds stresses in the near-wall region ((U)RANS region). $f_{e}$ is described as follows:

(11) $$f_{e}=max\left[\left(f_{e1}-1\right),0\right]\psi f_{e2}.$$

$f_{e1}$ solely is grid dependent, whereas $f_{e2}$ is a function of the flow field quantities. Further details regarding this methodology and related functions can be found in Shur et al. [5] and Gritskevich et al. [8].

As discussed, S-A IDDES and *k*-ω-SST IDDES employ the same triggering mechanism by introducing a modified length scale ($l_{IDDES}$) into the destruction term of $\tilde{\nu }$ (S-A IDDES) and *k* (*k*-ω-SST IDDES) transport equations. Therefore, the main focus of the present study is to investigate the effect of the underlying RANS model on overall model behavior when applied to different configurations ranging from fully-attached to separated flows.

## 3. Overview of the Test Cases

S-A IDDES and *k*-ω-SST IDDES are applied to two benchmark test cases, with increasing geometrical complexities. These include fully-developed turbulent channel flow and flow over a periodic hill. Various criteria/functions will be assessed on different grid resolutions to demonstrate the effect of near-wall modeling on model performance/behavior.

### 3.1. Turbulent Channel Flow

This test case will demonstrate the effect of underlying RANS model under stable and attached flow conditions. The size of the computational domain used for simulation is $L_{x}$ = 2π*h*, $L_{y}$ = 2h and $L_{z}$ = π*h*, where *x*, *y* and *z* denote the streamwise, wall-normal and the spanwise directions, respectively. Two friction Reynolds numbers have been considered; $Re_{\tau }$ = 395 and $Re_{\tau }$ = 4200, where $Re_{\tau }=\left(u_{\tau }*h\right)/\nu$ is based on friction velocity $u_{\tau }$ and channel half height *h*. Corresponding resulting mean Reynolds numbers based on the bulk mean velocity and channel half height are $Re_{b}$ ≈ 13,000 and $Re_{b}$ ≈ 200,000, respectively. Note that the computational domain is kept the same for all the considered Reynolds numbers. A constant pressure gradient is applied via the source term in the momentum equation to derive the flow at the required Reynolds number. Periodic boundary conditions are imposed in the streamwise and the spanwise direction, and the no-slip boundary condition is used for top and bottom walls. Three different grid resolutions (coarse, medium and fine) are used to simulate channel flow at $Re_{\tau }$ = 395. Constant geometric stretching of approximately 11% is used in the wall-normal direction. Please note that the resolution of the fine grid for $Re_{\tau }$ = 395 is equivalent to the DNS grid used by Moser et al. [11]. For $Re_{\tau }$ = 4200, two grid resolutions have been used. The coarse grid corresponds to the medium resolution used for $Re_{\tau }$ = 395 simulation. The fine grid is designed based on criteria to support transition to LES mode after the buffer layer and the beginning of the surface layer (i.e., $y^{+}$ ≥ 40–50), proposed by Brasseur and Wei [12]. This guarantees that the appropriate grid is available for transition from (U)RANS to LES mode at this Reynolds number, if the model functions properly. More details about the different grid resolutions used in this test case are summarized in Table 1. The results are compared with the DNS results of Moser et al. [11] (for $Re_{\tau }$ = 395) and Duran and Jiménez [13] (for $Re_{\tau }$ = 4200).

### 3.2. Periodic Hill Flow

**Figure 1:**
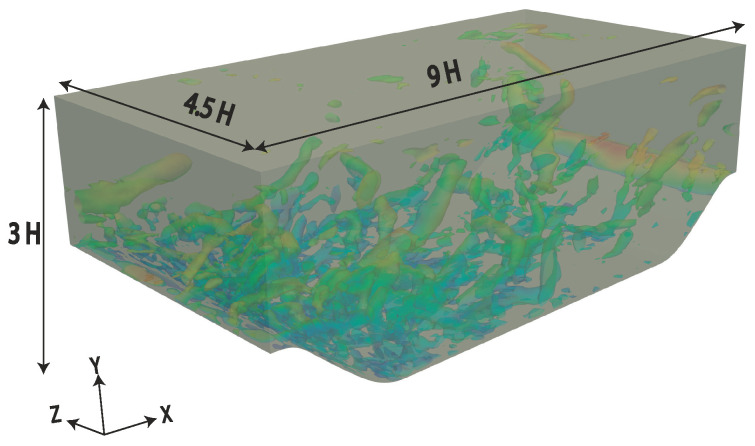
A three-dimensional geometry considered for periodic hill flow, with dimensions and the coordinate system employed in the present study. H, Height.

This is a typical test case to study separation and reattachment dynamics over a smooth curved surface. The size of the computational domain is 9H, 3H and 4.5H in the streamwise, wall-normal and spanwise directions, respectively, where H is the hill Height at the crest. The schematic of the flow domain is shown in Figure 1. Two bulk Reynolds numbers are investigated in the present study; $Re_{b}=$ 10,595 and $Re_{b}=$ 37,000, based on the hill Height (H) and the bulk velocity ($U_{b}$) at the crest. Two different grid-resolutions, similar to what is used in Razi et al. [14] to evaluate the PANS hybrid method, are used in this investigation (summarized in Table 2). Similar to the turbulent channel flow, the flow is driven by a constant pressure gradient, which is added as a source term in the streamwise momentum equation. Periodic inlet/outlet and spanwise boundary conditions were chosen, and therefore, the mean flow properties are also averaged in the spanwise direction. Results are compared with available experimental measurements [15] and high fidelity numerical simulation [16].

## 4. Quality Assessment

Hybrid (U)RANS-LES methods are increasingly applied to complex high Reynolds number flows relevant for industrial applications. In this context, assessment of the quality and reliability of hybrid model when operating in scale-resolving mode is essential. Several grid-based criteria have been proposed for LES quality assessment. These include Meyers et al. [17], Klein [18] and Celik et al. [19], to name a few.

In the present investigation, the scale-resolving region is assessed through various criteria summarized in Table 3. These will help in assessing the various capabilities of the IDDES methodology.

According to Pope [20], a well-resolved LES region can be defined when 80% of the total turbulent kinetic energy is resolved. Therefore, the ratio of modeled turbulent kinetic energy to total turbulent kinetic energy ($k_{modeled}/\left(k_{modeled}+k_{resolved}\right)$, Criterion 1 in Table 3), should attain its maximum (theoretically one) in the wall vicinity, where the model is supposed to operate in (U)RANS mode and should decrease to about 0.2 in the scale-resolving region of the simulation away from the wall. This criterion will also be used to evaluate the amount of intrusion of the scale-resolving region into the (U)RANS region. The intrusion could negatively affect the (U)RANS dynamics and ultimately near-wall prediction quality. Note that the modeled turbulent kinetic energy for S-A IDDES is calculated by relying on the Smagorinsky algebraic relation and is expressed as:

(12) $$k_{sgs.}={\left(\frac{\nu_{t}}{C_{s}\Delta }\right)}^{2}.$$

where $C_{s}$ ≈ 0.16 is obtained from Gong and Tanner [21].

The second criterion, related to the relative sub-grid scale viscosity, provides similar results as the first criterion in the scale-resolving region, therefore not shown in the present study.

The third criterion used in assessing grid resolution compares the characteristic cut-off length scale (▵, as defined in Equation (6)) to an estimated Kolmogorov length scale, (η), characterizing the length scale of the dissipative motion. In this criterion, η is obtained from the dissipation rate (ϵ) by the following relation:

(13) $$\eta ={\left(\frac{\nu^{3}}{\epsilon }\right)}^{1/4}.$$

where $\epsilon =\frac{{\left(k_{sgs.}\right)}^{3/2}}{L_{sgs.}}$. It should be noted that Equation (13) is merely a scale relation and provides a very conservative estimate of the finest scale in the turbulent flow. Considering a carefully-devised energy spectrum according to the Kolmogorov hypothesis, this criterion is particularly applicable to high Reynolds number flows [20]. However, Fröhlich et al. [22] applied this criterion to fairly low Reynolds number ($Re_{b}=$ 10,000) in wall-bounded flows and defined that the ratio should be around eight to ten in the scale-resolving region to resemble well-resolved LES. Furthermore, it demonstrates the significance of the sub-grid scale model to assess grid resolution requirement. In the present study, we assess this criterion only on channel flow with $Re_{\tau }=4200$ and periodic hill flow with $Re_{b}=$ 37,000.

The last criterion is based on the ratio of the sub-grid ($L_{sgs}$) and characteristic cut-off length scales. In *k*-ω-SST IDDES, the two-equation model is used to determine the sub-grid length scale ($k^{2}/\left(C_{\mu }\omega \right)$), whereas for S-A IDDES, the sub-grid length scale is a modified distance from the wall, $d_{w}$ ψ. The ratio of the sub-grid length scale and grid length scale should be of the same order in the scale-resolving region.

These criteria will be evaluated on the test cases discussed in the previous section. Importantly, these criteria are expected to respond appropriately to the grid refinement such that the scale-resolving mode will reflect the characteristics of the systematic eddy-resolving approach.

## 5. Numerical Procedure

All computations are performed using the open-source CFD code, Open-FOAM [23]. Two test cases, as described in Section 3, are investigated in the present study. In all cases, second-order central differencing for velocity, turbulent kinetic energy *k* and specific dissipation rate ω is used. The second-order time discretization method is used for all the simulations. Unsteady SIMPLE and PISO algorithms are used for momentum advancement and to solve the Poisson equation, respectively.

## 6. Results and Discussion

In this section, results obtained from *k*-ω-SST IDDES and S-A IDDES, when applied to channel flow and periodic hill flow will be presented and discussed. As mentioned before, the effect of near-wall modeling will be assessed on overall model prediction capability.

### 6.1. Turbulent Channel Flow

#### 6.1.1. $Re_{\tau }$ = 395

**Figure 2:**
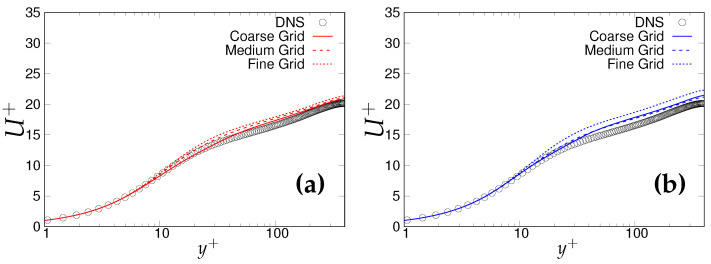
Channel flow at $Re_{\tau }=395$: wall normal variation of the non-dimensional velocity profile; (**a**) left column = *k*-ω-SSTIDDES; (**b**) right column = Spalart–Allmaras (S-A) IDDES.

**Figure 3:**
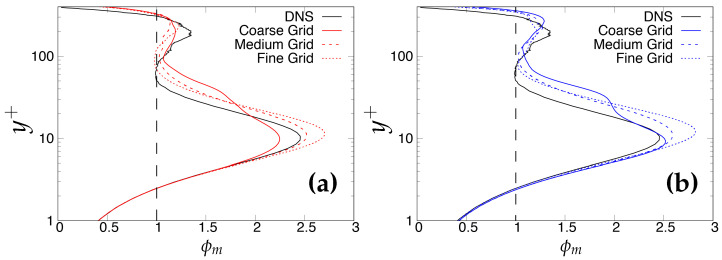
Channel flow at $Re_{\tau }=395$: variation of the normalized mean shear ($\phi_{m}$) as a diagnostic quantity for a log law. (**a**) left column = *k*-ω-SST IDDES; (**b**) right column = Spalart–Allmaras (S-A) IDDES.

Figure 2a,b shows non-dimensionalized velocity profiles obtained from three different grids under the two-equation (*k*-ω-SST IDDES) and one-equation (S-A IDDES) models, respectively. The log layer mismatch (LLM) in the outer log-layer is marginal in the case of the two-equation model, whereas it is observable for the one-equation model. It is expected that the LLM will become less and ultimately vanish at the DNS level mesh. However, both models and particularly S-A IDDES showed an inconsistent response to mesh refinement. To assess the LMM issue more accurately, the log-law indicator defined by Brasseur and Wei [12] was used. It is defined as the gradient of the mean streamwise velocity normalized by the inertial Law-Of-The-Wall (LOTW) surface-layer velocity and length scales:

(14) $$\phi \left(y\right)=\frac{y}{u_{\tau }}\frac{\partial \bar{U}}{\partial y},$$

where *y* and $u_{\tau }$ are the wall-normal distance and friction velocity at the wall. Figure 3 shows the variation of $\phi_{m}$ ($\phi_{m}$ ≡ κ ϕ(y)) plotted against wall-normal distance, where κ is the von-Karman constant, assumed to be 0.41 in the present study. According to the LOTW scaling, the log-law indicator should be constant and equal to one in the plateau region, which denotes the logarithmic region. However, it should be noted here that a true log-layer is not expected to appear, as $Re_{\tau }$ = 395 is too low [24]. Therefore, the main reason for accessing this quantity at $Re_{\tau }$ = 395 is to determine the discrepancy between models and DNS data in a more accurate manner. It is clearly shown that both models indicated an inconsistent behavior as the grid was further refined from a medium to a fine (DNS level) grid, i.e., deviation from DNS data became more noticeable.

**Figure 4:**
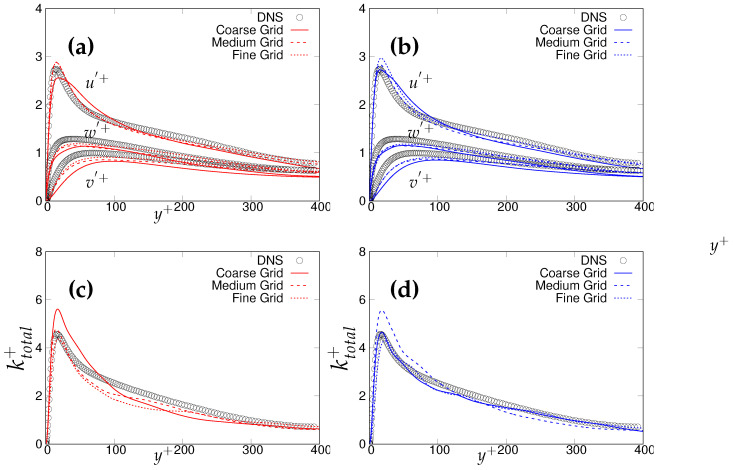
Channel flow at $Re_{\tau }=395$: wall-normal variation of: (**a**,**b**) resolved turbulence fluctuations; (**c**,**d**) non-dimensionalized total turbulent kinetic energy; symbols represent DNS. Left column = *k*-ω-SST IDDES, right column = Spalart–Allmaras (S-A) IDDES.

Further, we compare the resolved streamwise, wall-normal and the spanwise turbulent fluctuations in Figure 4a,b. In the vicinity of the wall, the fluctuations are well captured by the *k*-ω-SST IDDES model, and predictions improved in response to the grid refinement. In the S-A IDDES model, wall-normal and spanwise fluctuations were captured well in the near-wall region, whereas streamwise velocity fluctuation was over-predicted while progressing from the coarser to finer grid resolution. However, prediction of the resolved velocity fluctuations within both models was improved in the core region with advancement in grid resolution, signifying that the scale-resolving region responds appropriately to grid refinement at the present Reynolds number. Figure 4c,d shows the variation of total turbulent kinetic energy (resolved + modeled) along the wall normal direction. It can be observed that the k-ω-SST IDDES model responded more appropriately to grid refinement and the peak of total turbulent kinetic energy was well captured on the medium and fine (DNS level) grid. The inconsistent response of S-A IDDES might have been due to the existing uncertainties in calculating modeled turbulent kinetic energy. This is definitely a short-coming of S-A IDDES when sub-grid (modeled) quantities are important.

**Figure 5:**
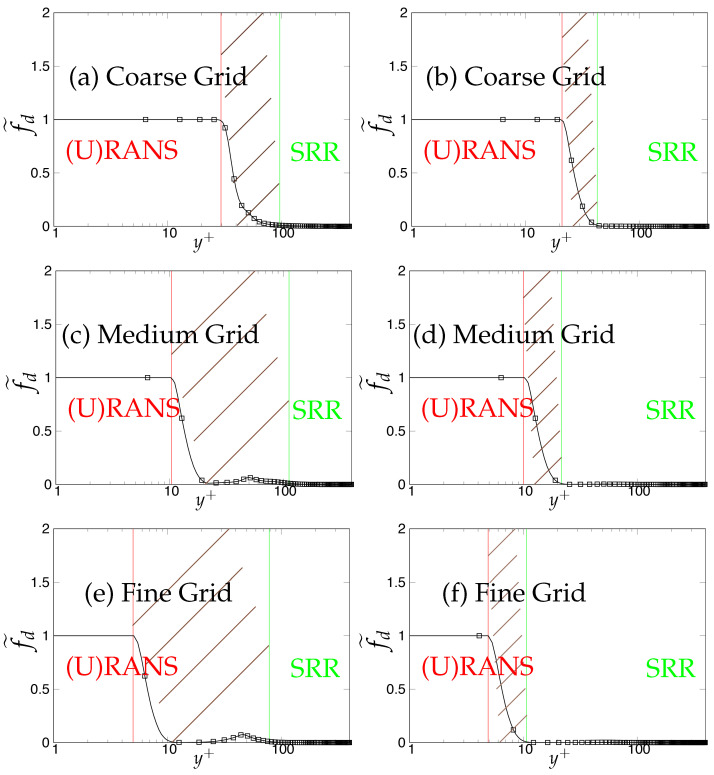
Channel flow at $Re_{\tau }=395$: response of $\tilde{f_{d}}$ to the grid refinement in *k*-ω-SST IDDES and S-A IDDES framework; (U)RANS = Unsteady RANS, SRR = Scale-Resolving Region and shaded region = grey area; left column = *k*-ω-SST IDDES, right column = Spalart–Allmaras (S-A) IDDES.

Later, we systematically investigate the effect of grid resolution on the main function ($\tilde{f_{d}}$) involved in the triggering mechanism and responsible for switching from (U)RANS to the scale-resolving mode. As mentioned previously, $\tilde{f_{d}}$ (also equals $max(1-f_{d},f_{B})$) defines the (U)RANS and scale-resolving region at $\tilde{f_{d}}=1$ and $\tilde{f_{d}}=0$, respectively. The grey region/area is defined in the range $0<\tilde{f_{d}}<1$ and is shown as the shaded region between (U)RANS and the Scale-Resolving Region (SRR) in the subsequent figures. The grid refinement should result in shrinkage of the grey region, as well as the (U)RANS region and ultimately vanishing on the finest (DNS level) grid. Figure 5a,c,e depicts the behavior of $\tilde{f_{d}}$ for the *k*-ω-SST IDDES model. As mentioned before, this function will help mainly to understand and evaluate the transition dynamics from (U)RANS to scale-resolving mode. Figure 5a,c,e shows that the grey area becomes larger while shifting towards the wall in response to grid refinement. This makes the (U)RANS region smaller, which is expected, as it allows the model to trigger to scale-resolving mode since the appropriate grid was provided. However, it is expected that similar to the (U)RANS region, the grey area would become smaller and allow the model to operate in scale-resolving mode in most part of the simulation on the finest grid.

In contrast to the two-equation model, the thickness of the grey area in S-A IDDES was not responsive to grid refinement and only was shifted towards the wall when the grid became finer. This behavior can be seen in Figure 5b,d,f. Comparing the results obtained from *k*-ω-SST IDDES and S-A IDDES may lead to the conclusion that improving the underlying RANS model does not necessarily improve the triggering mechanism and in fact might negatively affect it and lead to prolonged transition to scale-resolving mode due to the thicker grey region.

**Figure 6:**
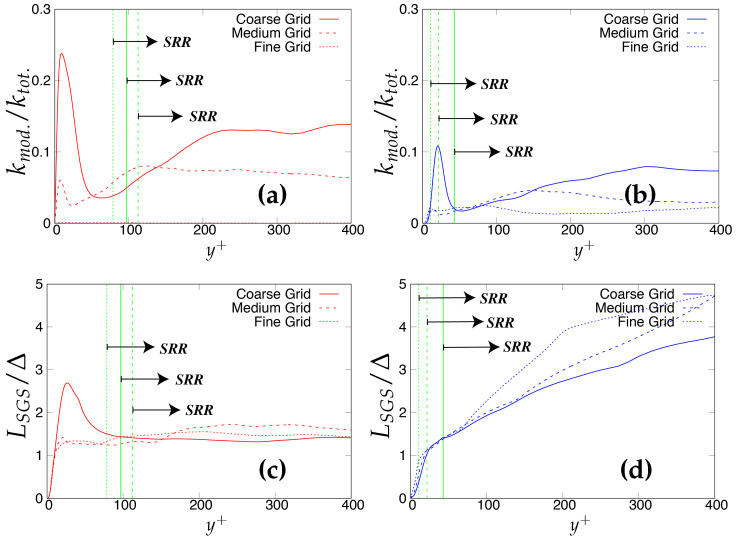
Channel flow at $Re_{\tau }=395$: variation of the ratio of (**a,b**) modeled to total turbulent kinetic energy and (**c,d**) integral length scale to characteristic cut-off length scale, along the wall normal direction; (U)RANS = Unsteady RANS, SRR = Scale-Resolving Region and shaded region = grey area; left column = *k*-ω-SST IDDES, right column = Spalart–Allmaras (S-A) IDDES.

**Figure 7:**
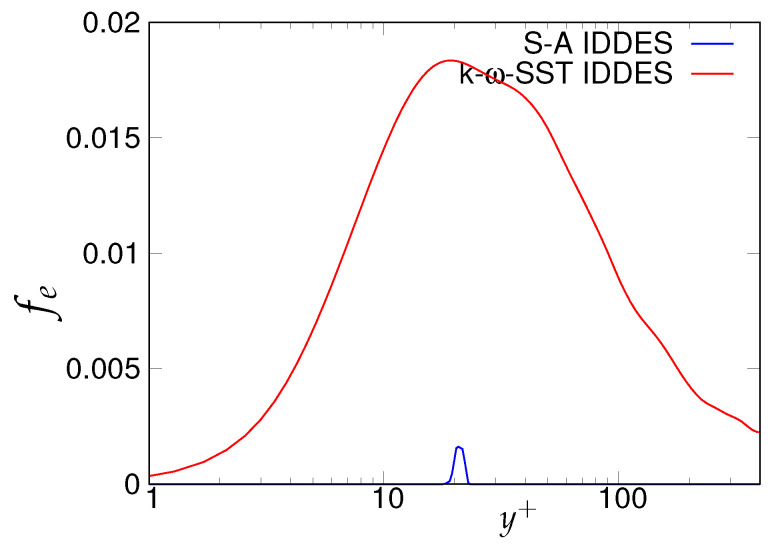
Channel flow at $Re_{\tau }=395$: response of elevating function ($f_{e}$) to the coarse grid resolution (64 × 192 × 48) in the *k*-ω-SST IDDES and Spalart–Allmaras (S-A) IDDES framework.

As the next step, the criteria stated in Table 3 will be assessed for determining the quality and reliability of the scale-resolving region. The arrows (shown in Figure 6) on the corresponding vertical lines towards increasing wall normal distance describe the Scale-Resolving Region (SRR). The ratio of the modeled to total turbulent kinetic energy (shown in Figure 6a,b) provides the extent of the modeled velocity scales, which should be around one in the wall vicinity in the case of coarse and medium grid. This ratio should become fairly negligible when the DNS level grid is used [20]. This is expected from both models and resembles the first criterion stated in Table 3. For both models, the ratio was significantly less than the one under the coarse and medium grid and reduced to nearly zero with the finest grid. This clearly shows a significant amount of intrusion from the scale-resolving region into (U)RANS part of the simulation that could be detrimental when near-wall effects need to be accurately modeled. The intrusion was due to the weak shielding provided to the underlying (U)RANS model and was confirmed by the variation of the function $f_{e}$, shown in Figure 7. As discussed in Section 2, function $f_{e}$ should provide necessary shielding to the near-wall (U)RANS region by preventing excessive reduction of the Reynolds stresses. Therefore, the behavior of $f_{e}$ under the *k*-ω-SST IDDES and S-A IDDES models on the coarse grid is compared in Figure 7. This function plays an integral role particularly when the IDDES methodology is applied to simulate high Reynolds number flows by preventing transition to scale-resolving mode when appropriate grid support is not available. Although shielding is stronger for the *k*-ω-SST IDDES model, which could be the reason for more amount of intrusion in S-A IDDES, it is not enough to prevent the intrusion. The $f_{e}$ went to zero under the medium and fine grid resolution.

Lastly, Figure 6c,d shows the variation of the ratio of the sub-grid length scale (obtained from underlying RANS model) and the characteristic cut-off length scale. The ratio should correspond to the same order in the scale-resolving region to represent the correct spectral dynamics on the energy spectrum. Both models satisfy the criterion in the core region. In the S-A IDDES model, the sub-grid length scale (distance from the wall) increases while traversing from the wall to the core of channel, and therefore, the ratio increases linearly and reaches its maximum at the channel center line while still preserving the correct spectral information.

The anisotropic behavior of the turbulence was analyzed through the “Lumley triangle [25]”. The Reynolds stress anisotropy tensor is defined by:

(15) $$b_{ij}=\frac{\langle u_{i}u_{j}\rangle }{\langle u_{k}u_{k}\rangle }-\frac{1}{3}\delta_{ij}.$$

where the trace of $b_{ij}$ is zero and departure from isotropy is defined between the two bounding lines, often represented in the ξ-η plane [20], where,

$$\xi ={\left(\frac{b_{ij}b_{jk}b_{kl}}{6}\right)}^{\frac{1}{3}},\;\;\;\eta ={\left(\frac{b_{ij}b_{ij}}{3}\right)}^{\frac{1}{2}}.$$

All physically realistic states of the turbulence should lie inside the triangle. The upper curve corresponds to the two-component turbulence, the left-hand curve to “axisymmetric contraction” and the right-hand curve to the “axisymmetric expansion”. The (0,0) point on the ξ-η plane corresponds to the isotropy point. Details can be found in Pope [20] and Sagaut [26].

**Figure 8:**
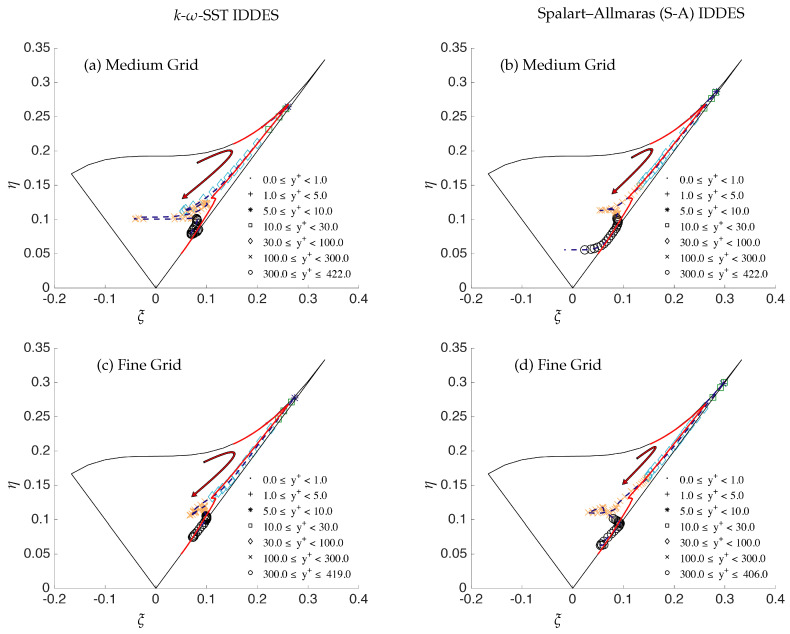
Channel flow at $Re_{\tau }=395$: anisotropy invariant map for three different mesh resolutions along the wall-normal direction; solid red line = DNS and points dash line = IDDES.

Figure 8 shows the anisotropy invariant map and compares the Reynolds stress structure obtained from S-A IDDES and *k*-ω-SST IDDES on the medium and fine grid. Furthermore, the behavior is compared with the DNS as it is expected that the fine (DNS level) grid should closely resemble the DNS profile obtained from Moser et al. [11]. Walking along the DNS profile, starting from the origin, we begin with an isotropic state for Reynolds stresses in the core region of the channel, moving forward to the small kink at $\Delta y^{+}$ ≈ 100, and then, the two-component turbulence state is achieved as we move close to the wall. The arrows shown in the Figure 8 point towards the core of the channel. The discrepancies in the Reynolds stress structure can be seen in the medium and fine grids, especially near the core of the channel for both models, which clearly demonstrate that it is independent of the underlying RANS model, and the IDDES methodology does not respond appropriately to grid refinement and may not be considered as a systematic eddy-resolving method.

#### 6.1.2. $Re_{\tau }=4200$

**Figure 9:**
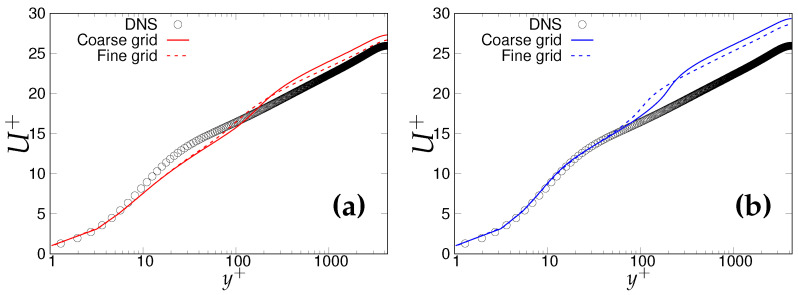
Channel flow at $Re_{\tau }=4200$: variation of the non-dimensionalized velocity profile along wall-normal direction: (**a**) left column = *k*-ω-SST IDDES, (**b**) right column = S-A IDDES.

**Figure 10:**
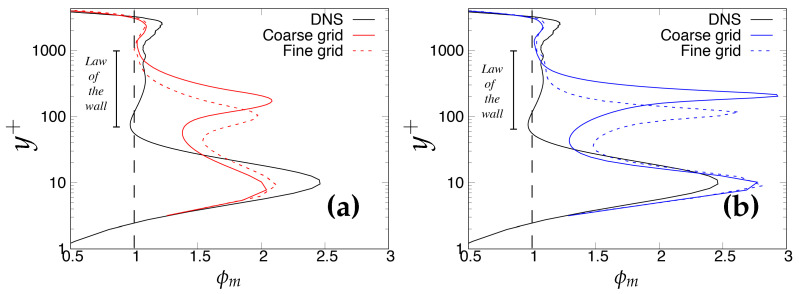
Channel flow at $Re_{\tau }=4200$: variation of the normalized mean shear ($\phi_{m}$) as a diagnostic quantity for a log law: (**a**) left column = *k*-ω-SST IDDES; (**b**) right column = S-A IDDES.

In this sub-section, effects of underlying RANS model on the overall behavior of IDDES methodology at higher Reynolds number are presented using a similar analysis approach as $Re_{\tau }$ = 395. Figure 9a,b demonstrates the mean velocity profiles obtained from the *k*-ω-SST IDDES and S-A IDDES models on coarse and fine grids. As can be seen, there was a clear overshoot in predictions of both models, which was more severe for the S-A IDDES model. Further, mesh refinement improved the situation only marginally. In order to show this more clearly, the log-law indicator ($\phi_{m}$) is plotted in Figure 10a,b. Deviation from the law of the wall (also called LMM) can distinctly be seen between starting from the upper part of the surface layer ($y^{+}$ ≈ 70) up to $y^{+}$ ≈ 1000, where $\phi_{m}$ should be close to unity. In addition, neither of the models were responsive to grid refinement, and non-significant improvement was observed when the grid became much finer. It may be deduced that the triggering mechanism of the IDDES methodology inappropriately responds to grid refinement at high Reynolds numbers.

**Figure 11:**
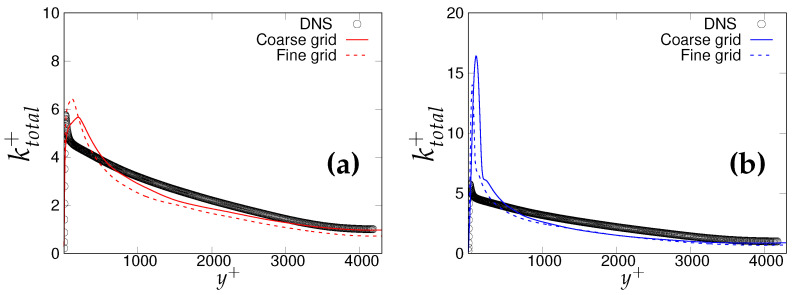
Channel flow at $Re_{\tau }=4200$: variation of non-dimensionalized total turbulent kinetic energy: (**a**) left column = *k*-ω-SST IDDES; (**b**) right column = S-A IDDES.

The wall-normal variation of non-dimensionalized total turbulent kinetic energy (modeled + resolved) and its response to grid refinement, for *k*-ω-SST IDDES and S-A IDDES model, is shown in Figure 11a,b, respectively. The peak of turbulent kinetic energy was pretty well captured by *k*-ω-SST IDDES in the wall vicinity, while S-A IDDES significantly over-predicted the peak magnitude. This was mainly due to the lack of an appropriate near-wall model in S-A IDDES that could lead to uncertainty in calculating the modeled turbulent kinetic energy. More importantly, the amount of uncertainty was considerably higher for a higher Reynolds number. In the core region, both models predicted the total turbulent kinetic energy fairly well, even on the coarse grid.

**Figure 12:**
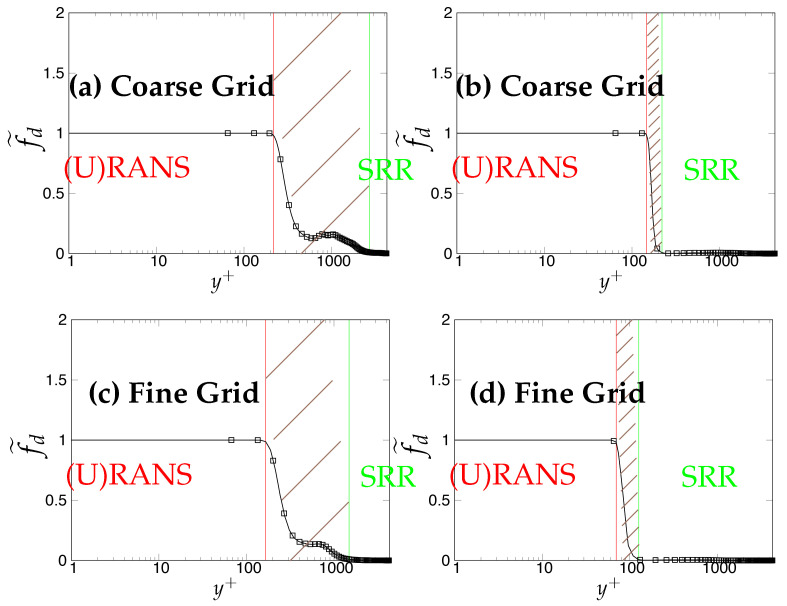
Channel flow at $Re_{\tau }=4200$: response of $\tilde{f_{d}}$ to the grid refinement in the kωSST IDDES and S-A IDDES framework; (U)RANS = Unsteady RANS, SRR = Scale-Resolving Region and shaded region = grey area; left column = *k*-ω-SST IDDES, right column = Spalart–Allmaras (S-A) IDDES.

The effect of underlying (U)RANS models and grid resolution on the dynamics of the grey area, depicted by the function $\tilde{f_{d}}$, is shown in Figure 12. For the coarse grid, shown in Figure 12a,b, the grey area ($0<\tilde{f_{d}}<1$) was significantly thinner in the case of the S-A IDDES model, compared to the one of the *k*-ω-SST IDDES model, indicating a delay in transition to scale-resolving mode, which might have been due to the more diffusive nature of the underlying RANS model in the *k*-ω-SST IDDES model. Further, the grey area in both models showed only slight sensitivity to grid refinement, as shown in Figure 12c,d. This may explain why grid refinement did not help to rectify the overshoot problem shown in Figure 9.

**Figure 13:**
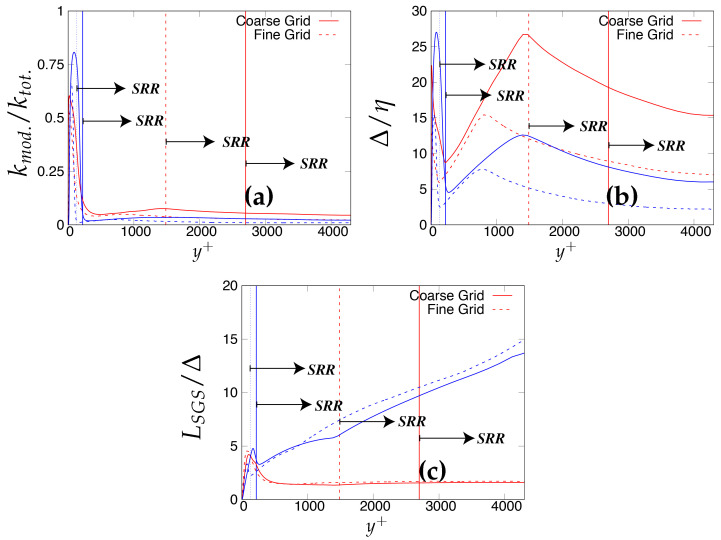
Channel flow at $Re_{\tau }=4200$: variation of the ratio of (**a**) modeled to total turbulent kinetic energy; (**b**) characteristic cut-off length scale to Kolmogorov length scale and (**c**) integral length scale to characteristic cut-off length scale, along the wall normal direction; SRR = Scale-Resolving Region; red = *k*-ω-SST IDDES, blue = Spalart–Allmaras (S-A) IDDES.

**Figure 14:**
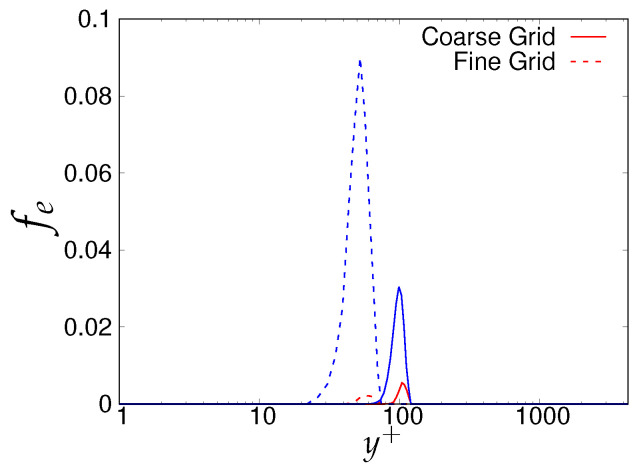
Channel flow at $Re_{\tau }=4200$: response of elevating function ($f_{e}$) to the different grid resolution in the *k*-ω-SST IDDES and S-A IDDES framework; red = *k*-ω-SST IDDES, blue = S-A IDDES.

The criteria stated in Table 3, for assessment of the scale-resolving region, are shown in Figure 13. The modeled the total turbulent kinetic energy ratio should attain a value of one (theoretically) in the wall vicinity, to prevent intrusion of the resolved scales from scale-resolving simulation to the (U)RANS solving simulation. The intrusion of resolved velocity scales in the (U)RANS region remained persistent under both models and could be observed from Figure 13a, showing around 25% and 50% of intrusion in the case of S-A IDDES and *k*-ω-SST IDDES, respectively. Unlike the $Re_{\tau }=395$ case, intrusion is less severe under both models. This can be attributed to the enhanced role of elevating function $f_{e}$, shown in Figure 14, which seems to be more responsive at a higher Reynolds number.

Figure 13b shows the variation of the ratio of the characteristic cut-off length scale and the Kolmogorov length scale for two different grid resolutions. For coarser grid resolution, the *k*-ω-SST IDDES model demonstrated that there was not appropriate grid support for LES, as the ratio in the core region was approximately one order of magnitude higher. In contrast, S-A IDDES satisfied the criterion, i.e., inaccurately confirmed appropriate grid support for LES. This clearly shows the relevance of sub-grid scale modeling to have an accurate assessment for grid resolution. By grid refinement, both models showed appropriate behavior in the core region and fell in the acceptable values to satisfy the criterion.

The last criterion, i.e., the ratio of the length scale provided by the underlying RANS model when operating in scale-resolving mode to the characteristic cut-off length scale, is shown in Figure 13c and should be of the same order in the scale-resolving region to satisfy the criterion. For S-A the IDDES model, an order increment in the fraction was seen in the core region. This was probably due to the length scale associated with the S-A IDDES model (distance from the wall), which increased while traversing from the wall to the core of the channel. It can be inferred that the S-A IDDES model did not satisfy this criterion at high Reynolds number, as the core region possessed the wrong spectral information. Therefore, it is hard to conclude that S-A IDDES switched to a true LES mode in the core region and could negatively impact the model prediction in the case of complex wall-bounded flows. In contrast, the *k*-ω-SST IDDES model satisfied this criterion as the ratio was of the same order in the core of the channel.

**Figure 15:**
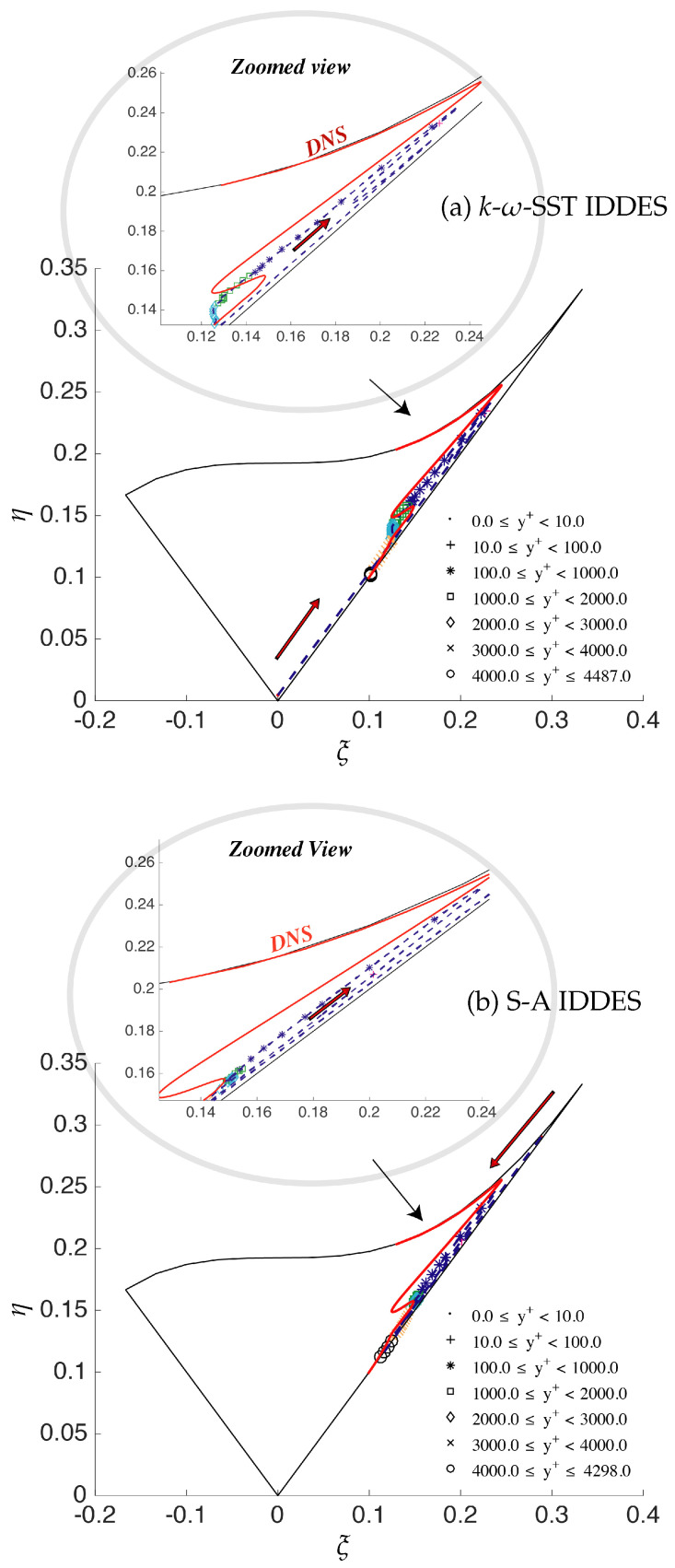
Channel flow at $Re_{\tau }=4200$: anisotropy invariant map of fine grid resolution along the wall-normal direction; solid red line = DNS and points and dashed line = IDDES.

The anisotropy behavior of the *k*-ω-SST IDDES and S-A IDDES models was analyzed qualitatively through the Lumley triangle, under fine grid resolution, and is shown in Figure 15a,b, where red colored arrows signify the starting point on the ξ and η plane. It can be seen that all Reynolds stress tensor invariants lied inside the boundaries of the Lumley triangle, which shows that the realizability constraint was well satisfied under both IDDES models. The effect of near-wall modeling (underlying RANS model) can distinctly be seen in the starting point in the Lumley triangle. In the case of the *k*-ω-SST IDDES model, the starting point resided in the three-component isotropic state, which is compatible with the RANS modeling assumption. However, due to the intrusion model, it was not able to retain it within the whole (U)RANS region, which was about $y^{+}\approx 100$. In contrast, S-A IDDES did not show any indication of RANS-like behavior, even very close to wall. Further, for both models, a tendency to reach the two-component turbulence state (completely opposite to DNS) was observed from $y^{+}$ ≈ 70 to $y^{+}$ ≈ 1000 and is shown in the zoomed view in Figure 15a,b. This region overlaps with the grey area for both models, which may indicate that inaccurate prediction of the dynamics of the grey area was contributing to this behavior. Moreover, this behavior confirms again that the near-wall model did not have much effect on overall model performance.

### 6.2. Periodic Hill Flow

The numerical modeling of flow separation around smoothly curved surfaces is challenging as compared to sharp-edge separation. This is mainly due to the prediction of a separation point or line, which is not fixed in space and is very sensitive to parameters like external flow properties, turbulence level, and development of a streamwise pressure gradient. The IDDES methodology, as one of the widely-used hybrid RANS-LES methods, is applied to periodic hill flow at two different Reynolds numbers. The main focus is to assess the capability of the method only to predict this flow with respect to the underlying RANS model.

#### 6.2.1. $Re_{b}=$ 10,595

**Figure 16:**
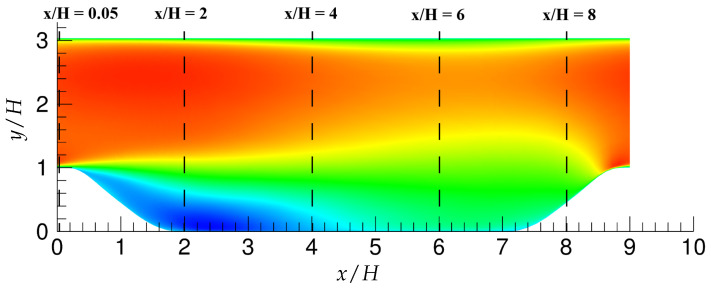
Periodic hill flow: considered streamwise locations, x/H = 0.05, 2, 6, and 8 for *Re_b_* = 10,000 and x/H = 0.05, 2, 4 and 8 for *Re_b_* = 37,000.

**Figure 17:**
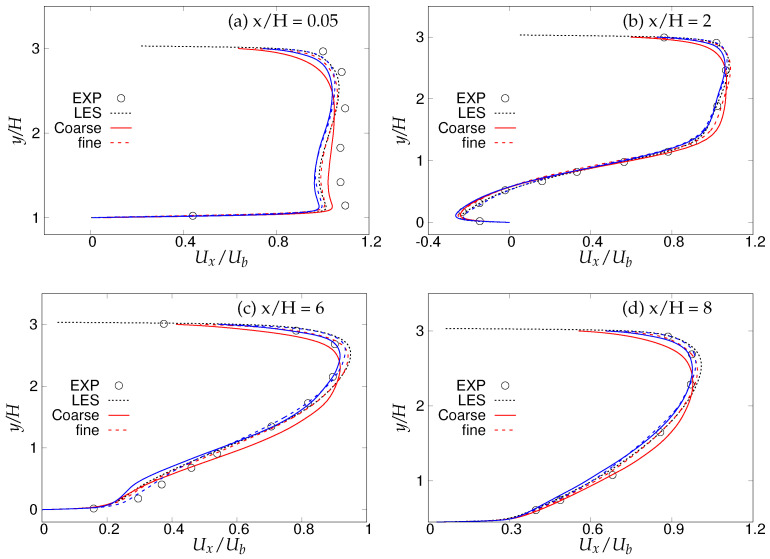
Periodic hill flow at $Re_{b}=$ 10,590: profiles of mean streamwise velocity at four different axial locations; red = *k*-ω-SST IDDES, blue = Spalart–Allmaras (S-A) IDDES.

Figure 16 shows different streamwise locations, i.e., x/H = 0.05,2,6, and 8, which are chosen for analyzing first and second order statistics, since these locations dictate the most critical physics in this flow configuration as reported in the experimental investigation of Rapp and Manhart [15] and the LES studies of Breuer et al. [16]. Note that the grid resolution used in LES prediction [16] is fine enough to resolve the near-wall dynamics. Figure 17 depicts the mean velocity profiles at the above-mentioned four streamwise locations. At x/H = 0.05, the flow acceleration in the lower wall vicinity was fairly well predicted by both models with marginal differences between fine and coarse grids. At the next location, x/H = 2, where the interaction of free shear layer separating from the crest of the hill and the reverse flow exists, mean streamwise velocity was well captured with moderate differences between fine and coarse grids. At x/H = 6, after reattachment, both models were able to capture the flow recovery from the low-energy separated region accurately on the fine grid. The flow started accelerating on the windward side of the hill at x/H = 8, and again, the behavior of both models lied between LES predictions and experimental measurements, with no significant sensitivity to grid refinement observed.

**Figure 18:**
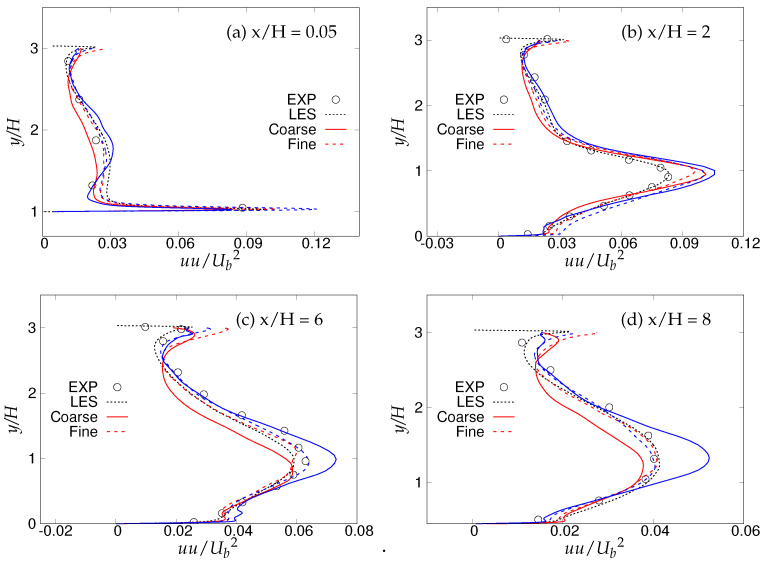
Periodic hill flow at $Re_{b}=$ 10,590: profiles of streamwise stress at four different axial locations; red = *k*-ω-SST IDDES, blue = Spalart–Allmaras (S-A) IDDES

**Figure 19:**
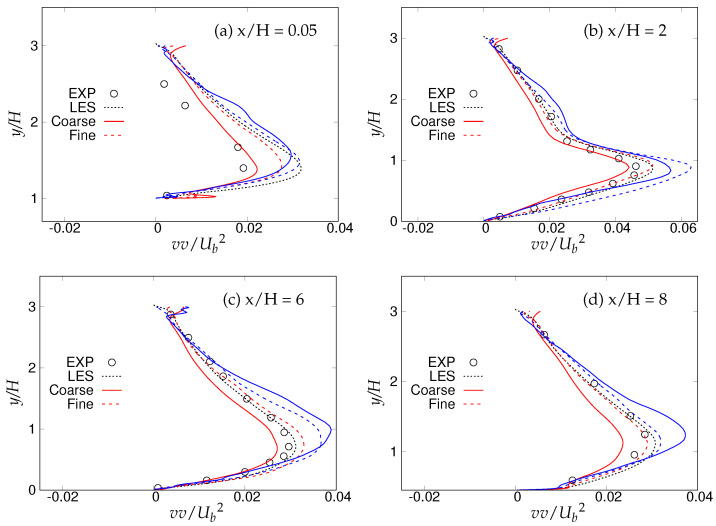
Periodic hill flow at $Re_{b}=$ 10,590: profiles of wall-normal stress at four different axial locations; red = *k*-ω-SST IDDES, blue = Spalart–Allmaras (S-A) IDDES.

**Figure 20:**
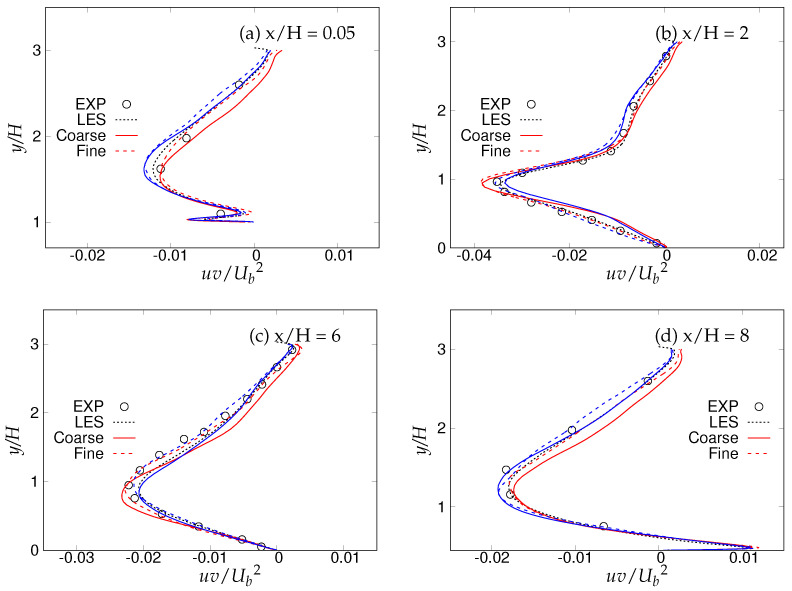
Periodic hill flow at $Re_{b}=$ 10,590: profiles of shear stress at four different axial locations; red = *k*-ω-SST IDDES, blue = Spalart–Allmaras (S-A) IDDES.

Figure 18, Figure 19 and Figure 20 show the streamwise, wall normal and shear stresses at the same locations. The total stress was computed as the sum of modeled and resolved stresses. Inside the recirculation zone, none of the models were able to capture the peak streamwise stress distribution, shown in Figure 18b. However, predictions were improved after flow reattachment, and results from both models were in fair agreement with LES predictions and experimental measurements. Further, a strong grid dependency on streamwise stresses could be seen after the reattachment location. The wall-normal stress profiles are shown in Figure 19. It can be seen that overall (except for x/H = 0.05), the *k*-ω-SST IDDES model delivered more accurate results, which showed more grid sensitivity compared to the streamwise stress. Discrepancies at the first location might have been due to the interference of the periodic boundary condition with model performance. Furthermore, a significant difference in the experiment [15] confirms the interference. Figure 20 depicts shear stress profiles at all four streamwise locations. Overall, good agreement with LES predictions and experimental measurements was observed for both models. Another important observation was that the shear stress in the vicinity of the lower wall remained the same within the S-A IDDES and *k*-ω-SST IDDES models, denoting insensitivity towards the near-wall model.

**Figure 21:**
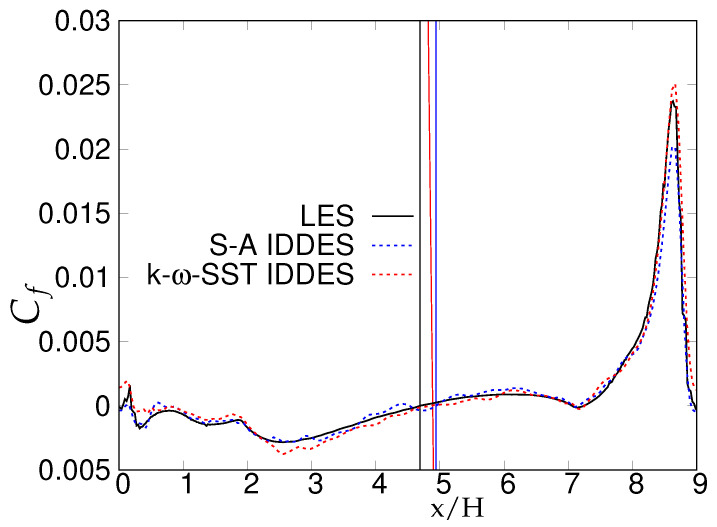
Periodic hill flow at $Re_{b}=$ 10,590: distribution of averaged skin friction coefficient for the fine grid; vertical lines denote the reattachment point.

Figure 21 shows the variation of skin friction coefficient over the lower wall-region for fine grid resolution. It can be seen that re-attachment locations predicted by both models were in a good agreement with both reference data, as shown in Table 4.

**Figure 22:**
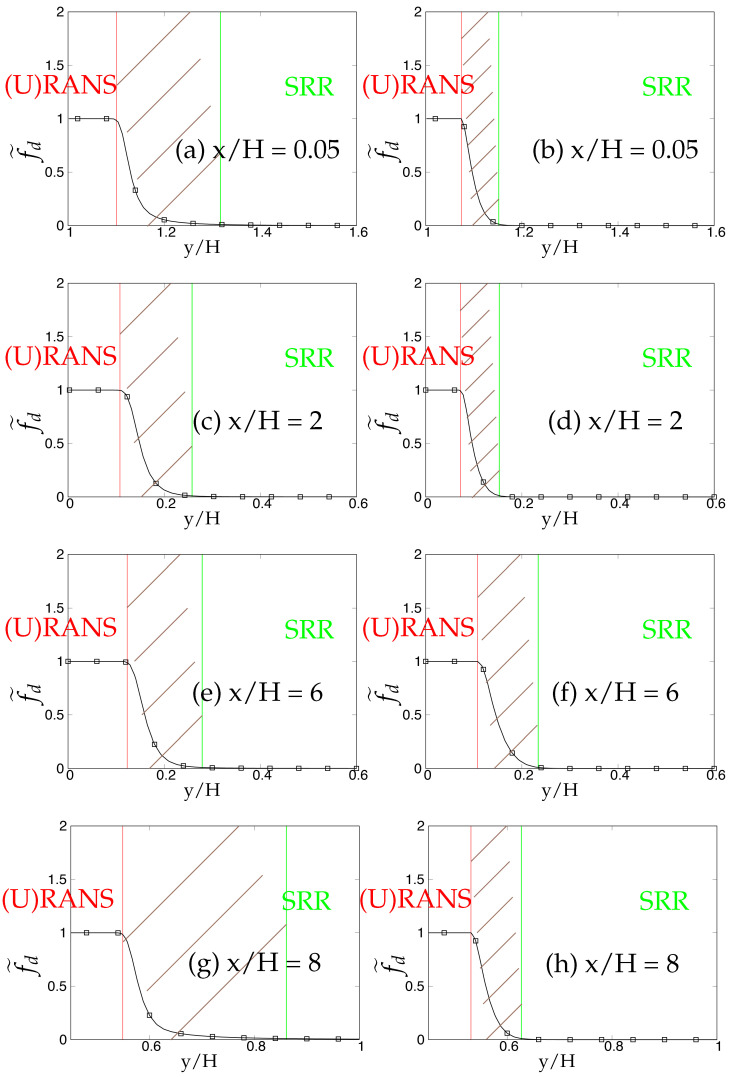
Periodic hill flow at $Re_{b}=$ 10,590: Response of $\tilde{f_{d}}$ to coarse grid resolution; (U)RANS = Unsteady RANS and SRR = Scale-Resolving Region; left column = *k*-ω-SST IDDES, right column = Spalart–Allmaras (S-A) IDDES.

**Figure 23:**
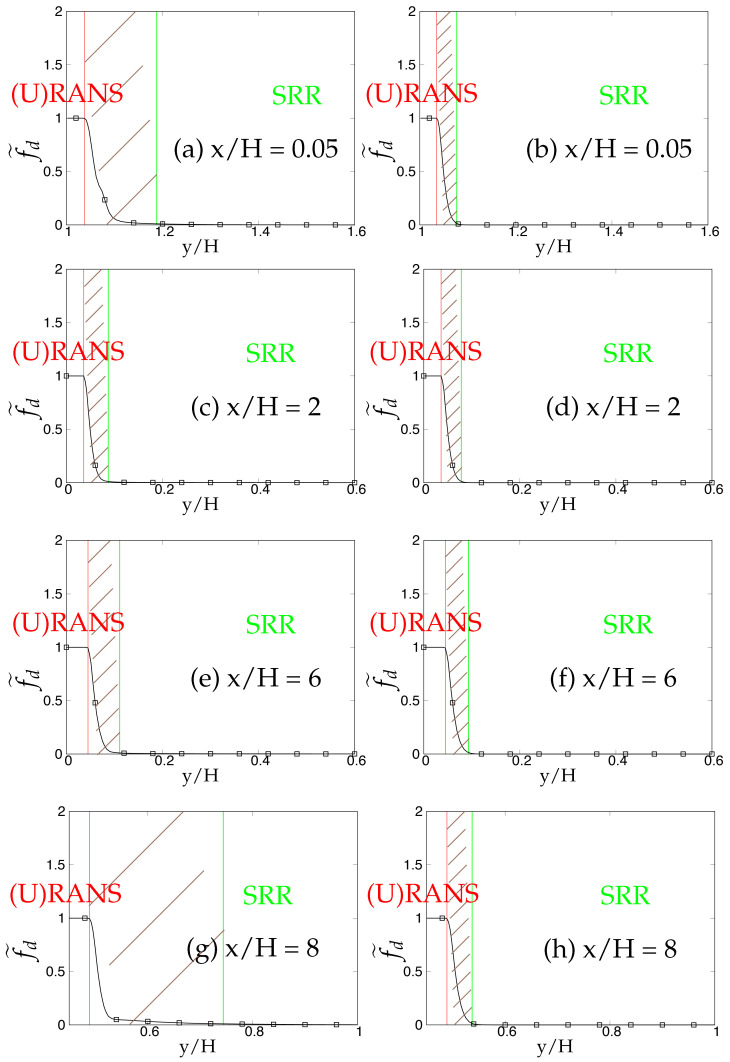
Periodic hill flow at $Re_{b}=$ 10,590: response of $\tilde{f_{d}}$ to fine grid resolution; (U)RANS = Unsteady RANS and SRR = Scale-Resolving Region; left column = *k*-ω-SST IDDES, right column = Spalart–Allmaras (S-A) IDDES.

**Figure 24:**
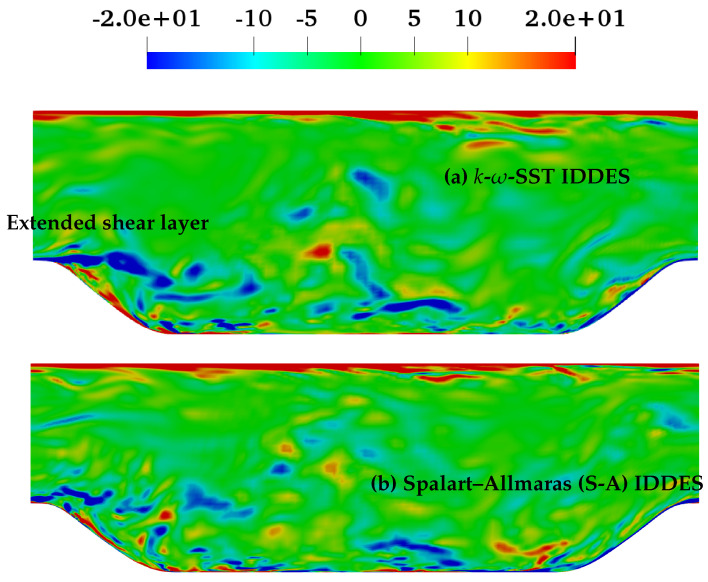
Periodic hill flow at $Re_{b}=$ 10,590: instantaneous field of spanwise vorticity for (**a**) *k*-ω-SST IDDES and (**b**) Spalart–Allmaras (S-A) IDDES, with fine grid resolution.

The behavior of the $\tilde{f_{d}}$ function responsible for transition from (U)RANS to scale-resolving mode under coarse and fine grids is shown in Figure 22 and Figure 23, respectively. In spite of shear layer instabilities emanating from the crest of the hill, the grey area predicted by the coarse grid resolution of *k*-ω-SST IDDES model was significantly larger than S-A IDDES. This might be an indication of the diffusive nature of the underlying RANS model in *k*-ω-SST IDDES. However, the grey area in S-A IDDES remained minimal at x/H = 0.05 and 2, signifying the strong influence of shear layer instabilities, and further downstream, broadened marginally at x/H = 6 and 8. For fine grid resolution, (U)RANS, as well as the grey region reduced significantly in the *k*-ω-SST IDDES model, but it was still considerably wider at locations where flow was attached, i.e., x/H = 0.05 and 8. However, for S-A IDDES, the grey area remained nearly constant at all four streamwise locations after grid refinement and only was shifted towards the lower wall. The insensitivity towards oncoming flow instabilities in the *k*-ω-SST IDDES model, under the fine grid resolution, could be seen from the extended attached shear layer convecting downstream from the crest of the hill, as shown in Figure 24a, whereas in the S-A IDDES model, the shear layer was highly unstable and may have been the reason for the swift transition to the scale-resolving mode.

**Figure 25:**
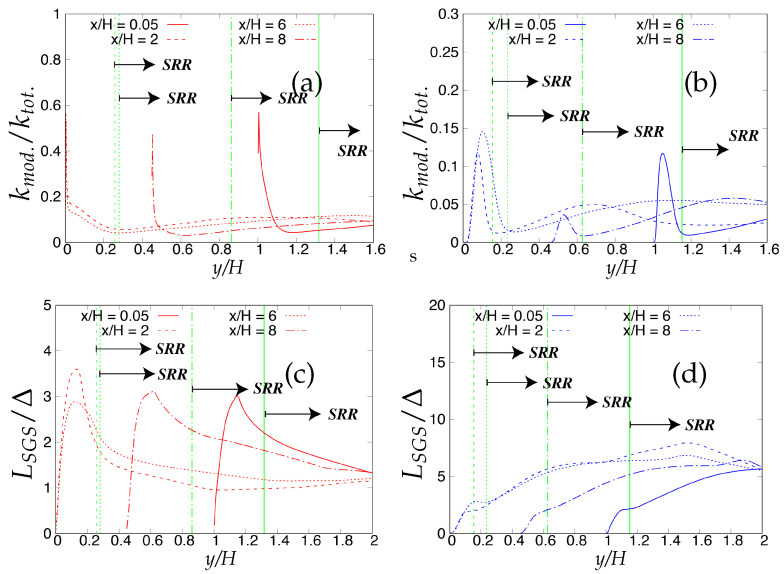
Periodic hill flow at $Re_{b}=$ 10,590: variation of the ratio of (**a**,**b**) modeled to total turbulent kinetic energy and (**c**,**d**) sub-grid length scale to characteristic cut-off length scale, along the wall normal direction under coarse grid resolution; SRR = Scale-Resolving Region; left column = *k*-ω-SST IDDES, right column = Spalart–Allmaras (S-A) IDDES.

Now, the criteria listed in Table 3 for assessing the quality and reliability of the scale-resolving region under different underlying (U)RANS models will be analyzed. Firstly, these criteria were applied to the coarse grid resolution and are shown in Figure 25. It can be seen from Figure 25a,b, where the ratio of modeled to total turbulent kinetic energy close to lower wall region signifies the intrusion from the scale-resolving region to the (U)RANS region is persistent among both models at all four streamwise locations. Similar to turbulent channel flow, the intrusion of the scale-resolved simulation could be attributed to the weak shielding of the (U)RANS region provided by the elevating function $f_{e}$. Further, from near-wall peak ratio, it is observed that the amount of intrusion for S-A IDDES was more severe as compared to *k*-ω-SST IDDES. As discussed in Section 4, the current Reynolds number is fairly low, and therefore, Criterion 3 in Table 3 was not applied in this section. The ratio of the sub-grid to characteristic cut-off length scale is shown in Figure 25c,d and is expected to be of the same order in the scale-resolving region to retain the spectral consistency. This criterion was satisfied by both models, depicting that the correct length scale is used in the scale-resolving mode by both models.

**Figure 26:**
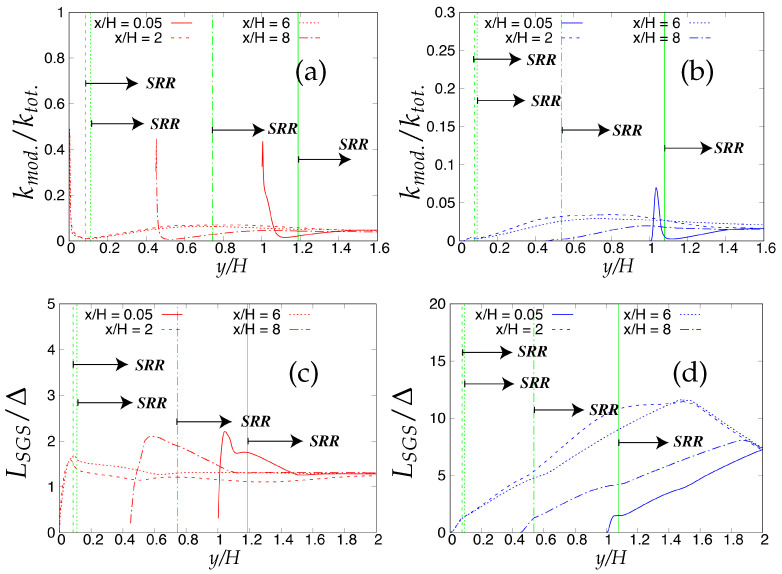
Periodic hill flow at $Re_{b}=$ 10,590: variation of the ratio of (**a**,**b**) modeled to total turbulent kinetic energy and (**c**,**d**) sub-grid length scale to characteristic cut-off length scale, along the wall normal direction under fine grid resolution; SRR = Scale-Resolving Region; left column = *k*-ω-SST IDDES, right column = Spalart–Allmaras (S-A) IDDES.

When applied to the fine grid resolution (shown in Figure 26), these criteria responded with different sensitivity under the given underlying (U)RANS model. Overall, intrusion of the scale resolving simulation into the (U)RANS region estimated from the ratio of modeled to total turbulent kinetic energy increased further with grid refinement. Similar to the coarse grid, it was more severe under the S-A IDDES model as compared to the *k*-ω-SST IDDES model.

The ratio of the sub-grid to characteristic cut-off length scale increased to one order magnitude higher at x/H = 2 and 6 for the S-A IDDES model and can be seen in Figure 26d. This outcome may allows us to conclude that in the S-A IDDES model, the characteristic length scale did not correspond to the appropriate sub-grid scale when the model was operating as a sub-grid scale model. In contrast, in the *k*-ω-SST IDDES model, the ratio remained at the same order at all four streamwise locations, confirming the appropriate length scale in scale-resolving mode.

**Figure 27:**
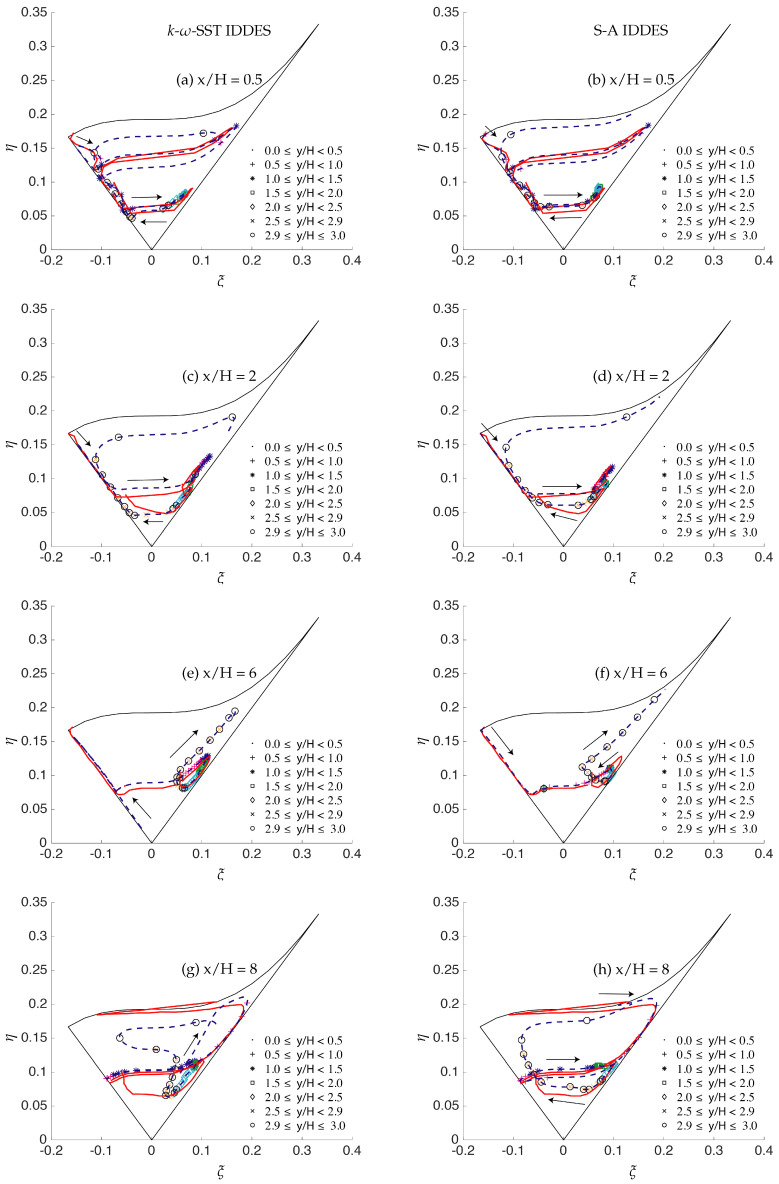
Periodic hill flow at $Re_{b}=$ 10,590: anisotropy invariant map for fine grid resolution along the wall-normal direction; solid red = well-resolved LES, points and dashed lines = IDDES.

Figure 27 shows the anisotropy invariant map at four streamwise locations in the flow direction for fine grid resolution. Important information about the anisotropic/isotropic states can be inferred from these plots when compared with highly resolved LES predictions [22]. First, all the data for the Reynolds stress tensor invariants lied inside the Lumley triangle, therefore satisfying the realizability constraint. Under S-A IDDES, the starting point was located at two-component turbulence state at all four streamwise locations. Up to x/H ≤ 6, the two-component turbulence state was achieved through the axisymmetric contraction line, while this approach was along the axisymmetric expansion line for x/H = 8. This behavior of the S-A IDDES model is consistent with the highly resolved LES findings of Fröhlich et al. [22]. However, the two-component turbulence state was never achieved within the *k*-ω-SST IDDES model while traveling towards the lower wall region from the core of the channel. At attached flow regions (x/H = 6 and 8), the Reynolds stress invariant map commenced from the near-isotropic line, i.e., the line crossing the point where ξ and η equal zero, indicating the underlying (U)RANS assumption of isotropic turbulence being well satisfied in *k*-ω-SST IDDES. The Lumley triangle provides qualitative assessment of flow anisotropy, while the quantitative measure of the Reynolds stress invariants can be assessed through the flatness parameter.

**Figure 28:**
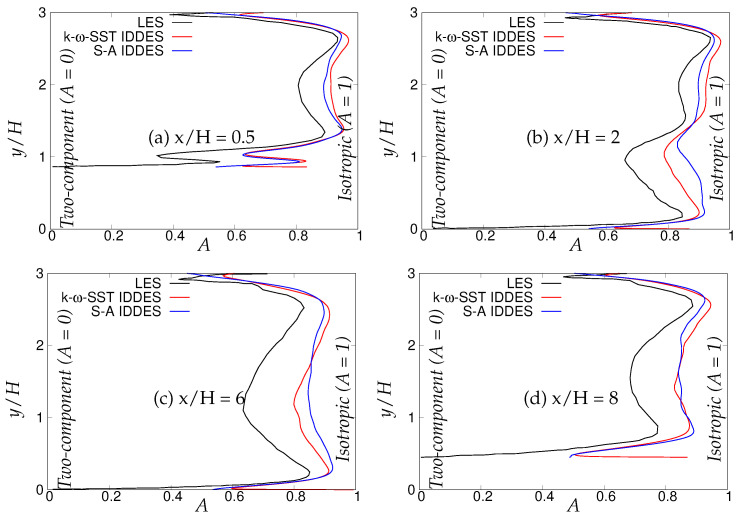
Periodic hill flow at $Re_{b}=$ 10,590: variation of the flatness parameter, *A*, at four streamwise locations.

The flatness parameter (*A*) is shown in Figure 28, also proposed by Lumley, combining Reynolds stress invariants using the following expression:

(16) $$A=1+9\left(\frac{b_{ij}b_{jk}b_{ki}}{3}-\frac{b_{ij}b_{ij}}{2}\right)$$

The value of *A* went to one for isotropic flow and defined the two-component turbulence state at *A* = 0. As shown in Figure 28, both models indicated more isotropic behavior away from solid walls compared to LES [22]. The flatness parameter (*A*) profiles for S-A IDDES depicted that Reynolds stress invariants have the tendency to reach the two-component turbulence state at the lower wall region. However, for the *k*-ω-SST IDDES model, the flow near the lower wall region closely resembled the isotropic state, which is consistent with assumption of isotropic turbulence in two-equation (U)RANS model concept. This clearly explains the effect of near-wall RANS model, which was shown to be more appropriate in the case of *k*-ω-SST IDDES, as the near-wall dynamics was supposed to be captured using the (U)RANS method.

#### 6.2.2. $Re_{b}=$ 37,000

We now proceed with examining the overall performance of the underlying (U)RANS model in response to the grey area and assessment of grid refinement for higher a bulk Reynolds number of 37,000. Indeed, at this Reynolds number, LES computations are prohibitively expensive and have not been performed in the literature. Therefore, the IDDES methodology will be evaluated using the experimental measurement provided by Rapp and Manhart [15].

**Figure 29:**
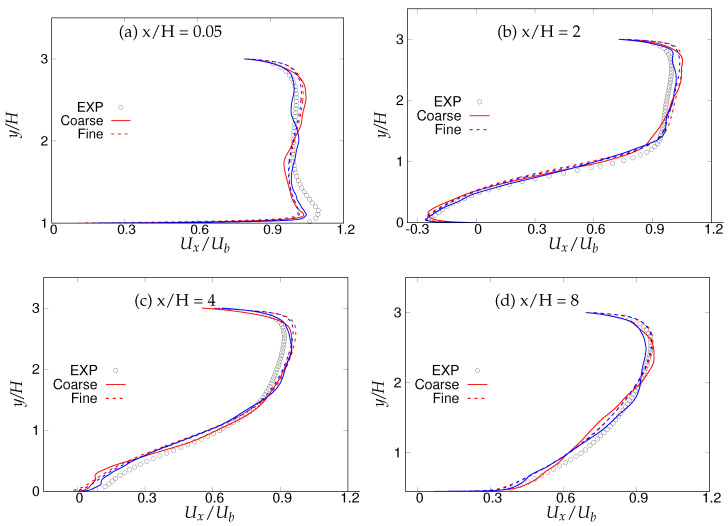
Periodic hill flow at $Re_{b}=$ 37,000: profiles of mean streamwise velocity at four different axial locations; red = *k*-ω-SST IDDES, blue = Spalart–Allmaras (S-A) IDDES.

We first investigate the effects of the underlying (U)RANS models and grid resolution on first-order flow statistics. The recirculation bubble became smaller with increasing Reynolds number [15]; therefore, we compared predictions with experimental measurements at streamwise locations (x/H) equal to 0.05, 2, 4 and 8, for this Reynolds number. Figure 29 shows the streamwise velocity profiles at four locations of x/H = 0.05, 2, 4 and 8 using coarse and fine grid resolutions. The flow acceleration close to the lower wall at x/H = 0.05 was under-predicted by both models, and prediction showed poor performance with increasing grid resolution. However, part of the disagreement might be due to the interference of the periodic boundary condition, as discussed before. The velocity profile in the wall vicinity was well predicted inside the recirculation region, at x/H = 2, by both models, with marginal discrepancies. According to experimental reference [15], the flow was expected to reattach at x/H = 4, but the velocity profile in the vicinity of lower wall at x/H = 4 (shown in Figure 29c) was under-predicted, which may have caused slow recovery from the upstream negative energy flow. x/H = 8 corresponds to the post-reattachment region, wherein the flow recovered from the upstream separated flow and the velocity profile was fairly well predicted by both models. Overall streamwise velocity predictions were in good agreement with experimental measurements with minor discrepancies.

**Figure 30:**
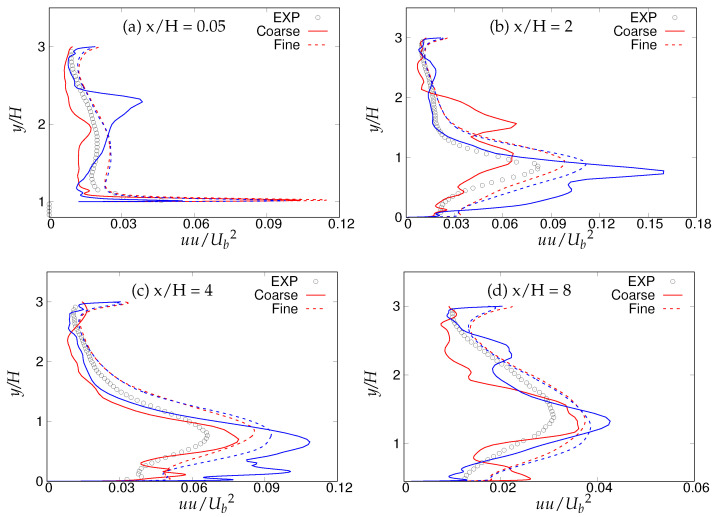
Periodic hill flow at $Re_{b}=$ 37,000: Profiles of streamwise stress at four different axial locations; red = *k*-ω-SST IDDES, blue = Spalart–Allmaras (S-A) IDDES.

**Figure 31:**
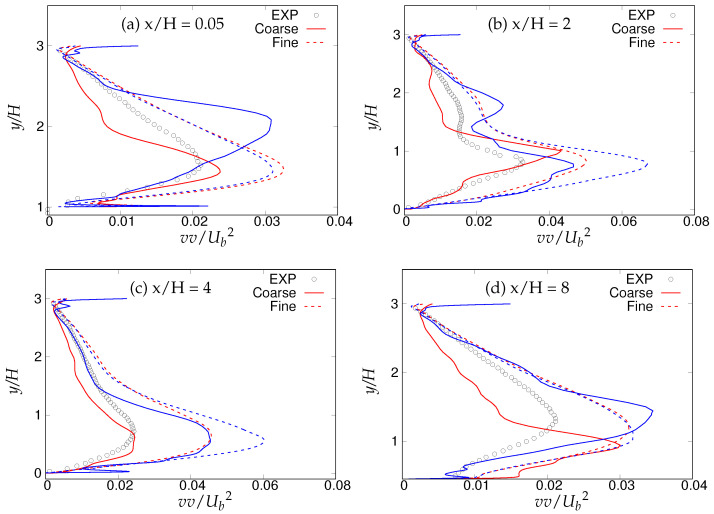
Periodic hill flow at $Re_{b}=$ 37,000: profiles of wall-normal stress at four different axial locations; red = *k*-ω-SST IDDES, blue = Spalart–Allmaras (S-A) IDDES.

**Figure 32:**
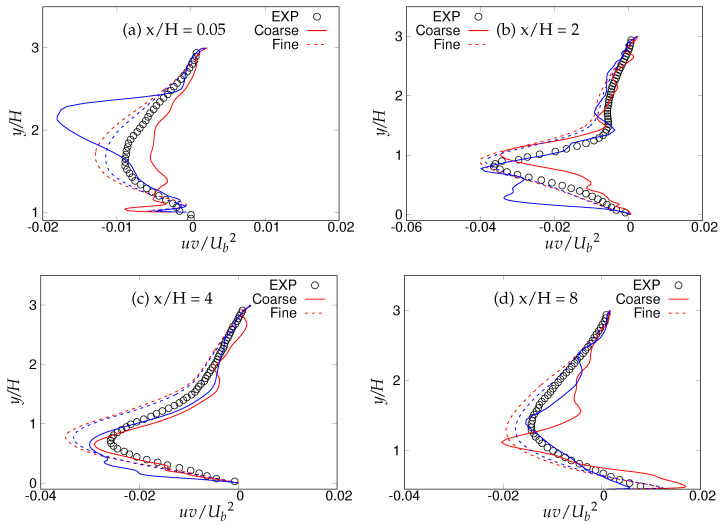
Periodic hill flow at $Re_{b}=$ 37,000: profiles of shear stress at four different axial locations; red = *k*-ω-SST IDDES, blue = Spalart–Allmaras (S-A) IDDES.

**Figure 33:**
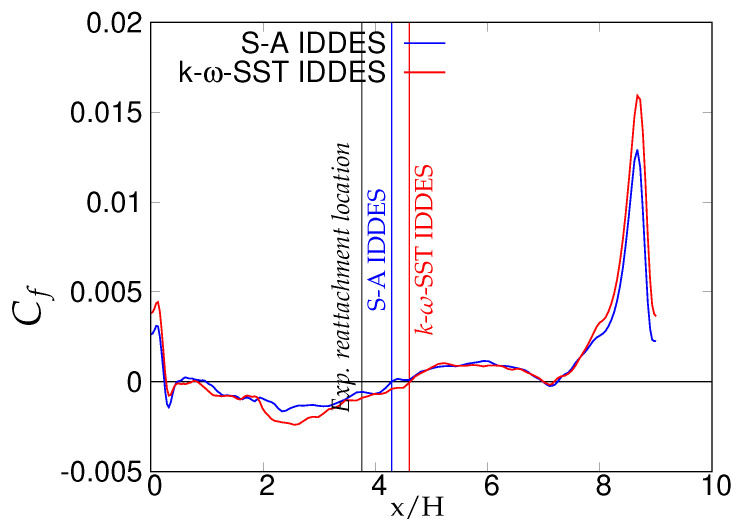
Periodic hill flow at $Re_{b}=$ 37,000: distribution of the averaged skin-friction coefficient for fine grid resolution; red = *k*-ω-SST IDDES, blue = Spalart–Allmaras (S-A) IDDES.

Figure 30, Figure 31 and Figure 32 show the components of the stress tensor at the four different streamwise locations. The effect of the grid sensitivity can be easily seen in the second-order statistics, where major discrepancies in the core region are shown under coarse grid resolution. Predictions obtained from the fine grid resolution showed the tendency to follow experimental measurements in the core region, however without an acceptable level of accuracy. The oscillating behavior of the results obtained on coarse grid, particularly in the near-wall region, may be indicative of detrimental effect of intrusion from the scale-resolving region into the (U)RANS region, allowing the model to resolve structures on a grid that is too coarse. This was more severe in the case of S-A IDDES, as the shielding function $f_{e}$ was weaker, as confirmed previously. This behavior strongly implies the negative effect of grid resolution on the IDDES methodology. The slow flow-reattachment process discussed earlier was confirmed by the plot of the friction coefficient shown in Figure 33, indicating a larger recirculation region predicted under the IDDES methodology. Unfortunately, at a higher Reynolds number of 37,000, the LES results were not available for quantitative comparison. Another interesting observation of this plot is the flow behavior right after the reattachment region. After reattachment and partial recovery from the negative energy flow, the flow appeared to be prone to separation at around x/H ≈ 7.5, where flow decelerated while moving towards the downstream hill, resulting in the local minimum in the friction coefficient plot. However, a further sharp rise in the friction coefficient was observed at the end of periodic hill.

**Figure 34:**
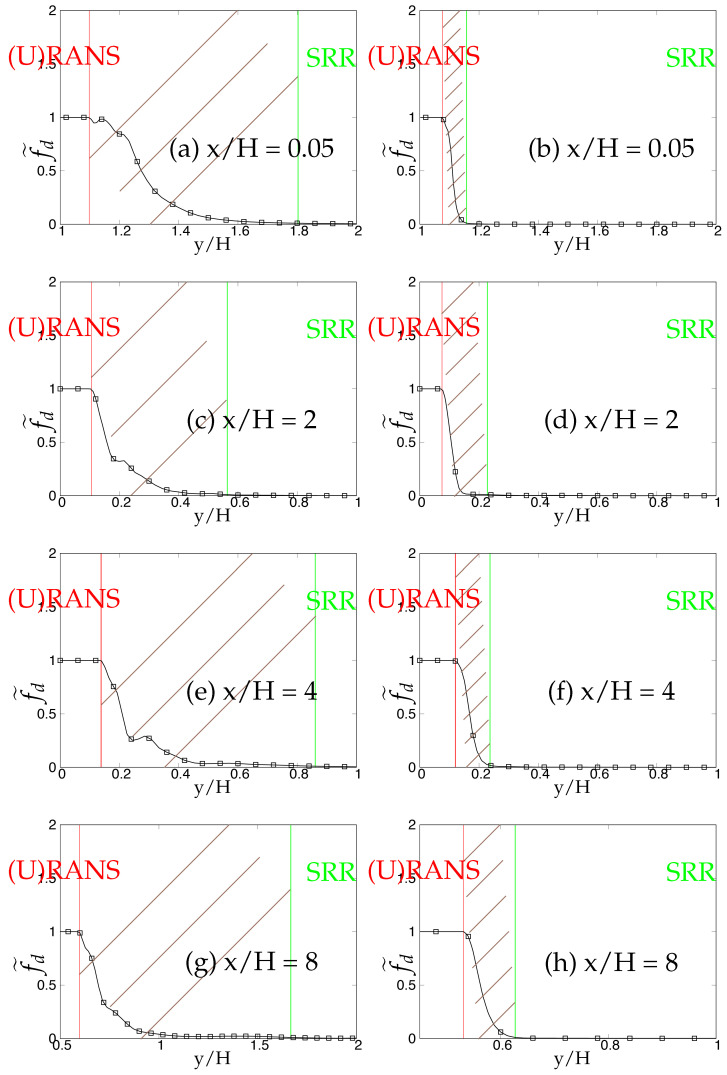
Periodic hill flow at $Re_{b}=$ 37,000: response of the $\tilde{f_{d}}$ function to coarse grid resolution; left column = *k*-ω-SST IDDES, right column = Spalart–Allmaras (S-A) IDDES.

**Figure 35:**
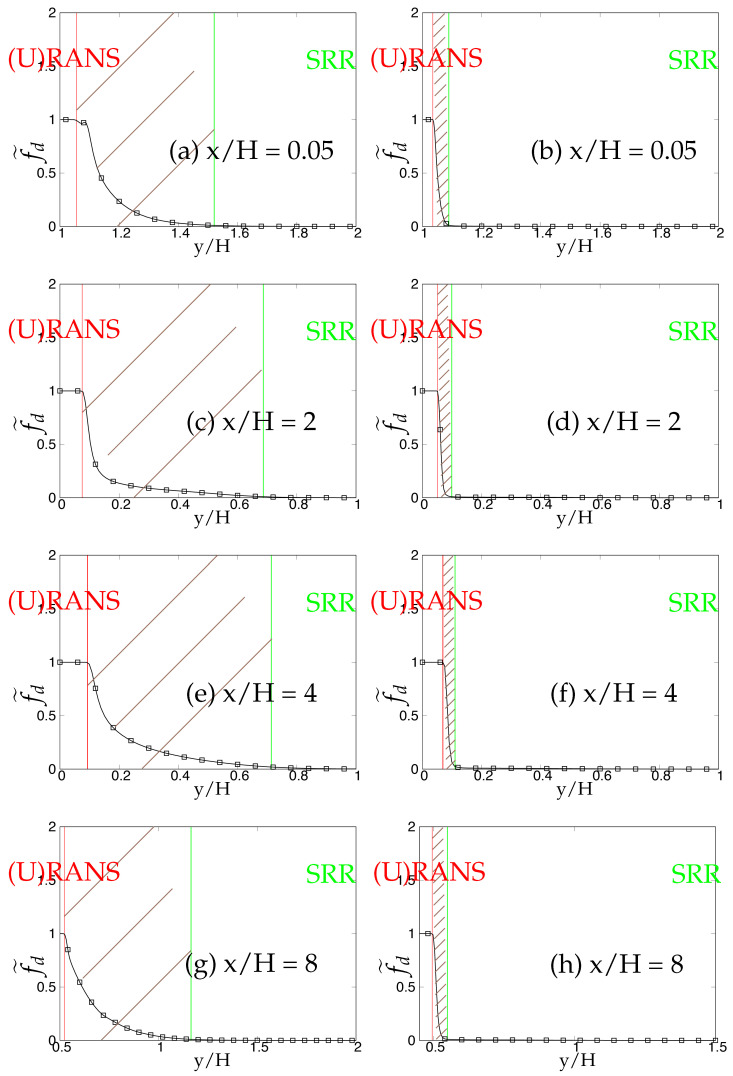
Periodic hill flow at $Re_{b}=$ 37,000: response of the $\tilde{f_{d}}$ function to fine grid resolution; left column = *k*-ω-SST IDDES, right column = Spalart–Allmaras (S-A) IDDES.

The dependency of the $\tilde{f_{d}}$ function on two different underlying (U)RANS models and grid refinement is shown in Figure 34 and Figure 35. At $Re_{b}=$ 37,000, the shear layer emanating from the crest of the hill was expected to be highly unstable and should have provided a substantial amount of flow instabilities to allow a swift transition from (U)RANS to the scale-resolving mode with a minimal grey area, especially in the recirculation region at x/H = 2. Within coarse grid resolution (shown in Figure 34), the grey region obtained at all four streamwise locations in the *k*-ω-SST IDDES model was significantly larger than the S-A IDDES model, indicating the prolonged transition from (U)RANS to scale-resolving mode in the *k*-ω-SST IDDES model. After grid refinement (shown in Figure 35), the grey area was extended within the *k*-ω-SST IDDES model at x/H = 2 and 4, demonstrating the inconsistent behavior of the model. It is concluded that the grey area obtained from the IDDES methodology using the advanced underlying (U)RANS model did not respond appropriately to grid refinement and in fact further grew with increasing Reynolds number under separated flows. On the other hand, the grey area in the S-A IDDES model after grid refinement stayed nearly constant or insensitive and only shifted spatially towards the decreasing wall-normal distance, which was not an expected outcome in the case of systematic-eddy-resolving simulation.

**Figure 36:**
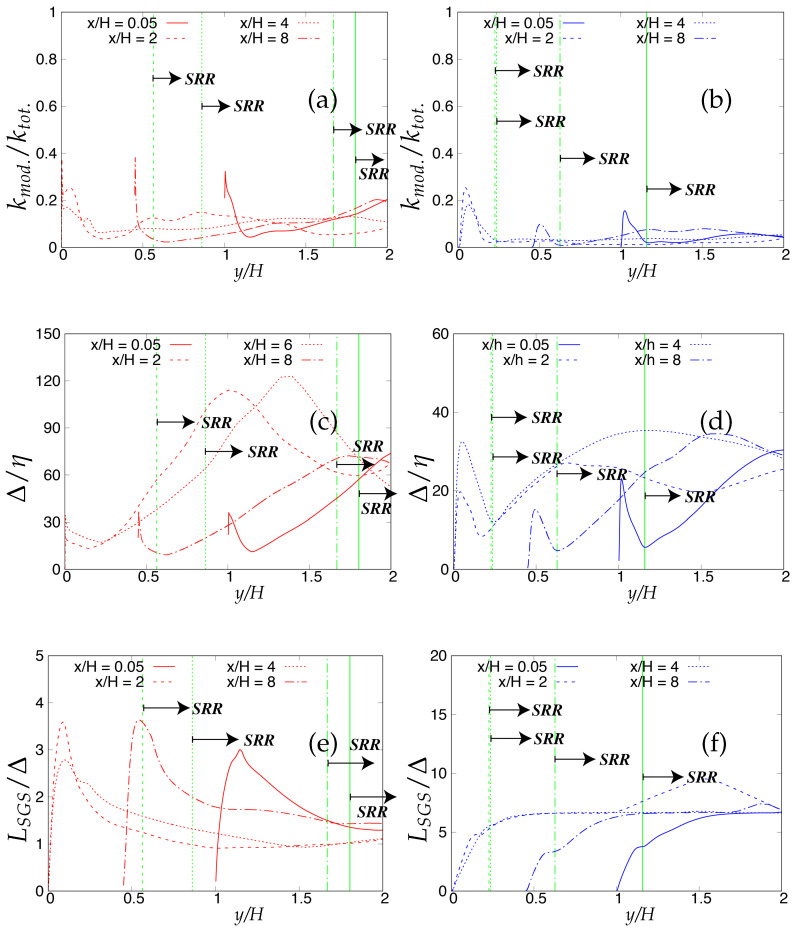
Periodic hill flow at $Re_{b}=$ 37,000: variation of the ratio of (**a**,**b**) modeled to total turbulent kinetic energy; (**c**,**d**) the characteristic cut-off length scale to the Kolmogorov length scale and (**e**,**f**) the sub-grid length scale to characteristic the cut-off length scale, at four different streamwise locations under coarse grid resolution; SRR = Scale-Resolving Region; left column = *k*-ω-SST IDDES, right column = Spalart–Allmaras (S-A) IDDES.

In the next step, the criteria listed in Table 3 were investigated on coarse and fine grid resolutions, subsequently. As one of the major issues we have seen so far, the intrusion of scale-resolving simulation into the (U)RANS simulation could be seen from the ratio of modeled to total turbulent kinetic energy in coarse grid resolution under both models, shown in Figure 36a,b. The average amount of intrusion was around 60% and 75% in the *k*-ω-SST IDDES and S-A IDDES models, respectively. While comparing with the periodic hill flow case at $Re_{b}=10,000$, it is observed that the shielding effect seemed more responsive for S-A IDDES at higher Reynolds number, resulting in a lesser amount of intrusion, whereas the opposite trend was seen under the *k*-ω-SST IDDES model at a higher Reynolds number. This behavior under different underlying (U)RANS models was consistent with the channel flow case at higher Reynolds number.

Further, the ratio of the characteristic cut-off length scale and Kolmogorov length scale for coarse grid is shown in Figure 36c,d. First of all, the maximum peak ratios signify that the most dissipation occurred inside the scale-resolving region, and next, the level of ratios in the core region compares the accuracy of sub-grid scale models used in each model. However, calculation states that the current grid resolution was coarse for LES/scale-resolving simulation in the scale-resolving region. During the sub-grid scale operation mode under the coarse grid, both models satisfied the third criterion in Table 3, i.e., the ratio of sub-grid length scale and the characteristic cut-off length scale, shown in Figure 36e,f, was of the same order at all four streamwise locations.

**Figure 37:**
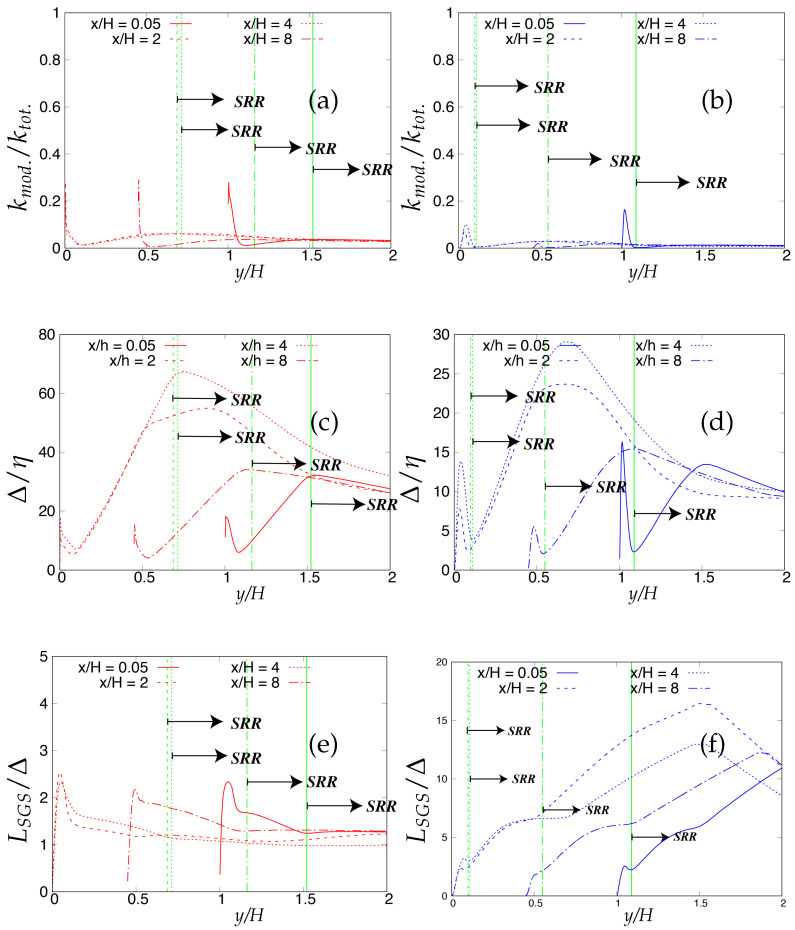
Periodic hill flow at $Re_{b}=$ 37,000: variation of the ratio of (**a**,**b**) modeled to total turbulent kinetic energy; (**c**,**d**) the characteristic cut-off length scale to the Kolmogorov length scale and (**e**,**f**) the sub-grid length scale to the characteristic cut-off length scale, at four different streamwise locations under fine grid resolution; SRR = Scale-Resolving region; left column = *k*-ω-SST IDDES, right column = Spalart–Allmaras (S-A) IDDES.

Under fine grid resolution, the severity of the intrusion of scale-resolved simulation into (U)RANS simulation could be seen from the ratio of modeled to total turbulent kinetic energy in Figure 37a,b, especially in the S-A IDDES model. The variation of the ratio of the characteristic cut-off length scale and Kolmogorov length scale is shown in Figure 37c,d. It is important to note here that the current grid resolution was extremely coarse to address the well-resolved LES region at such a high Reynolds number. For *k*-ω-SST IDDES, the ratio in the scale-resolving region indicated that grid support was not sufficient for LES simulation at all four streamwise locations, whereas for S-A IDDES, the level of the ratio falsely satisfied the criterion while traversing to the core region, as the current grid resolution is not fine enough for well-resolved LES simulation. Comparing the ratios in the *k*-ω-SST IDDES and S-A IDDES models demonstrates the importance of sub-grid scale modeling to have an accurate assessment for grid resolution. An inappropriate sub-grid length scale to characteristic length scale was obtained in the S-A IDDES model, as shown in Figure 37f. Like the turbulent channel flow discussed in the previous section of channel flow at $Re_{\tau }$ = 4200, a higher Reynolds number flow defies the systematic-eddy-resolving approach in this case, as well, mostly due to the length scale associated with the S-A IDDES model, which is simply the distance from the wall.

Based on the results obtained from the channel flow and periodic hill flow configuration, we have found that choosing the more advanced underlying (U)RANS model for accurate modeling of near-wall dynamics was insensitive to the flow instabilities under the IDDES methodology. Therefore, a significant amount of grey area was obtained using the advanced underlying (U)RANS model, while the grey area obtained from the simple (U)RANS model, i.e., one-equation model, remained minimal and of nearly constant thickness at different locations in the flow. Overall, it can be concluded that irrespective of grid resolution, neither models showed the characteristics of being the systematic-eddy-resolving approach in the scale-resolving region.

## 7. Summary and Conclusions

The IDDES methodology using two different underlying (U)RANS models to capture near-wall dynamics has been applied to two distinct benchmark test cases: channel flow and periodic hill flow, at two different Reynolds numbers. The main focus was to investigate the effect of the near-wall model on overall model prediction capability, the dynamics of the grey area and the response to grid refinement. It turns out that near-wall model does not have any significant effects on the prediction of first and second order statistics. Further, it was shown that using an advanced underlying (U)RANS model (*k*-ω-SST IDDES) provides an extended grey region compared to the one-equation model (S-A IDDES), resulting in a delayed transition to the scale-resolving mode, which might be due to the diffusive nature of the two-equation model (*k*-ω-SST IDDES).

Moreover, inconsistent responses of the grey area to grid refinement were observed for both models, such as not vanishing when the DNS level grid was used within both models or getting extended (particularly for *k*-ω-SST IDDES). Furthermore, it was observed that there is a Reynolds number dependency in the response of the grey area to grid refinement, generally with more inconsistency at higher Reynolds numbers in the case of *k*-ω-SST IDDES. In contrast, the grey area in S-A IDDES is thinner, indicates slight sensitivity regarding grid refinement and, generally, shifted towards near-wall when the grid becomes finer.

Inconsistent behavior may suggest that the dynamics of the grey area (responsible for allowing transition from (U)RANS to scale-resolving mode) cannot be captured using empirical blending functions mostly dominated by geometrical parameters rather than flow field quantities.

Three different criteria have been applied to assess the reliability and quality of the scale-resolving region within the IDDES methodology. First, intrusion of the scale-resolving simulation into (U)RANS simulation has been observed within both models, which is inappropriate, as the grid design in the near-wall region is not viable to support scale-resolving simulation and, therefore, may result in inaccurate modeling of near-wall dynamics. This can be seen in the oscillatory behavior of the statistics for periodic hill flow at $Re_{b}$ = 37,000 when the coarse grid is used. Secondly, S-A IDDES falsely satisfies the third criterion (stated in Table 3) and reports well-resolved LES simulation residing in the core region even in the case when the grid is too coarse. Conversely, the *k*-ω-SST IDDES model correctly satisfies the criterion under both benchmark test cases. This clearly shows the relevance of sub-grid scale modeling to have an accurate assessment for grid resolution. Regarding the last criterion, at higher Reynolds number flows, the S-A IDDES model when operating in sub-grid scale operation mode cannot be considered as true LES.

D’Alessandro et al. [27] compared the S-A IDDES model to a DES methodology built on a non-linear (U)RANS model and a $k-\epsilon -v^{2}$ based model [28] when applied to separated flow (there is no significant difference between the IDDES and DES methodology in separated flows [5]). Results did not show any significant difference among the above-mentioned models.

Results presented in this study along with other observations mentioned before may lead to the conclusion that improving the underlying (U)RANS model alone would not significantly improve the capabilities of the IDDES. Further, they suggest that the main reason for the observed shortcomings in the IDDES methodology mostly likely are due to inaccurate predictions of the grey area. Further progress will require additional focus on capturing the dynamics of the grey area accurately to make this methodology a reliable tool that can be applied to various flow configurations at different Reynolds numbers and grid resolutions.

## Figures and Tables

**Table 1:** Details of the grid resolution for turbulent developed channel flow.

Reτ	Grids	Δ $x^{+}$	Δ $y_{w}^{+}$	Δ $z^{+}$	$N_{x}$	$N_{y}$	$N_{z}$
395	Coarse	41.60	0.1	27.7	64	192	48
	Medium	20.84	0.1	13.90	128	192	96
	Fine	10.04	0.09	6.56	256	192	196
4200	Coarse	212.2	1.03	140.2	128	192	96
	Fine	117.6	1.09	57.9	234	146	234

**Table 2:** Details of the grid resolution for periodic hill flow.

Reb	Grid	$N_{x}$	$N_{y}$	$N_{z}$	$N_{\mathrm{total}}$
10,595 and 37,000	Coarse	100	100	30	300,000
	Fine	150	100	60	900,000
LES for 10,595 [16]	-	-	-	-	11,300,000

**Table 3:** List of grid assessment criteria for the scale-resolving region in the present study.

Equation	Criterion	Description
(1)	$k_{resolved}$/($k_{resolved}$+$k_{sgs}$)	ratio of the resolved turbulent kinetic energy to the total turbulent kinetic energy, where resolved turbulent kinetic energy is defined as $k=\frac{1}{2}\langle {u^{{}^{\prime }}}_{i}{u^{{}^{\prime }}}_{i}\rangle$ and modeled turbulent kinetic energy is defined as Equation (12).
(2)	$1/(1+0.05{\left(\frac{\nu +\nu_{t}}{\nu }\right)}^{0.53})$	relative sub-grid scale viscosity ratio
(3)	Δ/η	ratio of the characteristic cut-off length scale to the relative Kolmogorov length scale
(4)	$L_{sgs.}$/Δ	the ratio of the sub-grid length scale and the characteristic cut-off length scale

**Table 4:** Dependency of grid-resolution on the reattachment location.

Cases	Modeling Framework	Grid-Resolution	${\left(x/h\right)}_{\mathrm{Reattach}.}$
$Re_{b}=$ 10,000	One-equation model	100 × 100 × 30	4.8172
		150 × 100 × 60	5.021
	Two-equation model	100 × 100 × 30	4.6072
		150 × 100 × 60	4.9166
Fröhlich et al. (2005)	LES		4.6
Rapp and Manhar (2011)	Experimental location		4.21
$Re_{b}=$ 37,000	One-equation model	100 × 100 × 30	3.9578
		150 × 100 × 60	4.2922
	Two-equation model	100 × 100 × 30	3.9578
		150 × 100 × 60	4.7078
Rapp and Manhar (2011)	Experimental location		3.76
